# Nurses’ knowledge, attitudes, and practices in pressure ulcer prevention in intensive care units: associations with burnout

**DOI:** 10.7717/peerj.21250

**Published:** 2026-05-14

**Authors:** Mahmoud Abdel Hameed Shahin, Samia Eaid Elgazzar, Rasha Mohammed Hussien, Mohamed Gamal Elsehrawy

**Affiliations:** 1 Nursing Department, Prince Sultan Military College of Health Sciences, Dhahran, Eastern Region, Saudi Arabia; 2 Department of Medical–Surgical Nursing, College of Nursing, Qassim University, Buraydah, Qassim, Saudi Arabia; 3 Department of Community, Psychiatric and Mental Health Nursing, College of Nursing, Qassim University, Buraydah, Qassim, Saudi Arabia; 4 Nursing Administration and Education Department, College of Nursing, Prince Sattam Bin Abdulaziz University, Al-Kharj, Saudi Arabia; 5 Nursing Administration Department, Faculty of Nursing, Port Said University, Port Said, Egypt

**Keywords:** Attitude, Burnout, Knowledge, Nurses, Pressure ulcer

## Abstract

**Background:**

Nurses play a crucial role in preventing pressure ulcers, as they are at the forefront of patient care and are responsible for implementing preventive measures. The current study aimed to explore nurses’ knowledge, attitudes, and practices regarding pressure ulcer (PU) prevention in Intensive Care Units (ICUs) and to identify any correlation between these variables and nurses’ burnout.

**Methods:**

This study was conducted in the intensive care units of Zagazig University Hospitals in Egypt, with data collection from January 2024 to May 2024. The study included 131 nurses working in these units. Data were collected using a self-administered questionnaire that included sociodemographic information, nurses’ knowledge, attitudes, and practices related to pressure ulcer prevention, and a burnout assessment tool.

**Results:**

Nurses exhibited moderate knowledge of pressure ulcer prevention (16.1 ± 4.6). Nurses demonstrated moderately favorable attitudes overall, with strong agreement on key prevention principles (*e.g.*, the avoidability of PUs and the need for risk assessments), but perceived challenges with time and prioritization. However, adherence to recommended practices was low, with 54.8% of checklist items not completed. Additionally, these nurses experienced a notable level of burnout, particularly in cognitive and emotional domains (3.6 ± 0.8 and 3.6 ± 0.9, respectively). A significant, moderate positive correlation was found between nurses’ knowledge of pressure ulcer prevention and their practices (*r* = 0.353, *p* < 0.001).

**Conclusion:**

ICU nurses demonstrated moderate knowledge and a relatively positive, moderate attitude toward pressure ulcer prevention. However, they also identified several potential obstacles to the effective implementation of preventive practices. ICU nurses experienced very high levels of burnout, particularly in cognitive and emotional domains. The knowledge and attitudes of ICU nurses concerning pressure ulcer prevention emerged as essential factors influencing their clinical practices.

## Introduction

Pressure ulcers (PUs) are a significant health concern affecting an estimated three million people worldwide (Au et al., 2019; Mervis & Phillips, 2019). These ulcers are described as localized injuries to the skin or underlying tissues, generally over a bony prominence, that arise from pressure alone or in combination with shear or friction (Au et al., 2019; Bereded, Salih & Abebe, 2018; Mervis & Phillips, 2019; Moore & Patton, 2019). According to Khojastehfar, Najafi Ghezeljeh & Haghani (2020), nurses are responsible for maintaining patients’ skin integrity and preventing pressure ulcers while patients are in the hospital. Therefore, nurses must possess a comprehensive understanding of the pressure ulcer (PU) scales to determine patients who may be vulnerable and take appropriate preventive action. This not only alleviates patient discomfort but also shortens hospital stays and reduces the risk of long-term complications, such as deep tissue damage that can manifest months after superficial healing (Morehead & Blain, 2014). PUs are linked to substantial mortality, usually due to sepsis that occurs after bacterial inoculation of the bloodstream through the ulcer. PUs cause prolonged morbidity due to pain and related psychological effects.

Knowledge serves as the foundation for practice, resulting in high-quality, secure healthcare. Most global research demonstrated that nurses lack sufficient knowledge and have unsuitable attitudes in this area of interest (Coventry et al., 2024; Etafa et al., 2018; Grešš Halász et al., 2021). In the last 15 to 20 years, not much has changed about this phenomenon. The Pieper’s Pressure Ulcer Knowledge Test (PUKT) was employed in multiple studies to assess nurses’ knowledge of pressure ulcer therapy. Previous studies examined registered nurses’ understanding of pressure ulcers. Participants’ scores increased with the recentness of their reading about pressure ulcers. Age, educational attainment, or years of nursing experience were not related to the knowledge (Pieper & Mott, 1995). Studies have revealed a lack of knowledge among critical care nurses in recognizing and preventing pressure ulcers. The knowledge was not found to be influenced by years of nursing experience, type of nursing school attended, or current interest in reading papers about pressure ulcer therapy. Moreover, few nurses have studied the recommendations for preventing pressure ulcers (Pieper & Mattern, 1997). It is often observed that nurses have poor adherence to pressure ulcer prevention guidelines, which may be attributed to their limited knowledge (Wu et al., 2022). In the study by Claudia et al. (2010), nurses who had completed a structured 7.5- hour pressure ulcer training program, those working in medical and nephrology wards, and those who perceived their own knowledge as higher achieved significantly better knowledge scores regarding pressure ulcer prevention and treatment. These findings indicate meaningful differences in both risk- related variables (*e.g.*, sector of employment, level of perceived knowledge) and preventive strategies (*e.g.*, adherence to risk assessment using the Braden Scale and implementation of preventive interventions), underlining the importance of targeted education and work environment in improving evidence- based pressure ulcer prevention practice.

Burnout is a complex psychological condition brought on by ongoing stress at work. Feelings of tiredness or low energy typify it, a distancing of the mind from one’s work or cynicism, and a decrease in professional efficacy (Baliyan, 2024). Burnout is gaining attention as a primary public health problem for healthcare workers’ (HCWs’) mental health issues (Søvold et al., 2021). It puts patient safety at risk, particularly the quality of treatment provided (Hodkinson et al., 2022), including in PU care.

Intensive care units, or ICUs, are small organizations where effective knowledge management is essential to producing positive outcomes. ICU patients are at particularly high risk of pressure ulcers due to immobility, hemodynamic instability, and technological dependency, making systematic, nursing-led preventive strategies such as repositioning and mobilization protocols essential components of ICU care (Korompeli et al., 2025). At the same time, these intensive preventive demands increase nurses’ workload and cognitive burden, which may contribute to job burnout, especially when staffing or organizational support is insufficient (Gündüz & Öztürk, 2025). By framing ICUs as knowledge-intensive environments where effective acquisition, interpretation, and application of clinical information are required to prevent pressure ulcers. In acquiring knowledge, data are found initially, followed by information (data interpretation), and finally, knowledge (Indrasiene et al., 2021; Suárez-Gonzalo & Catalá-López, 2018).

Furthermore, because of the social ties arising from the care process and the duality of health and sickness, the knowledge necessary for nursing practice is as complicated as the nursing profession (Indrasiene et al., 2021). This sophistication emphasizes the importance of knowledge management to guarantee excellent patient care and successful PU prevention measures. This complexity underscores the importance of effective knowledge management to ensure high-quality patient care and effective PU prevention strategies. This study aimed to explore nurses’ knowledge, attitudes, and practices regarding pressure ulcer prevention in Intensive Care Units and to identify any correlations among those variables and job burnout among nurses.

## Materials & Methods

### Study design

A descriptive-correlational design was used, and data were collected from a single study group at the same time. The descriptive–correlational design was selected because it is appropriate for exploring existing levels of nurses’ knowledge, attitudes, and practices regarding pressure ulcer prevention and for examining their relationships with job burnout within a single population at a single point in time. The study also adhered to Strengthening the Reporting of Observational studies in Epidemiology (STROBE) guidelines. This ensures that all key elements of research- study design, methods, results, and limitations- are reported clearly and transparently, which improves the internal and external validity of the manuscript, facilitates critical appraisal by reviewers and readers, and enhances reproducibility and comparability with other studies.

### Setting and sample

This research study was conducted in the intensive care units of Zagazig University Hospitals in Egypt, and data collection took place from January 2024 to May 2024. Using the open-source software OpenEpi and considering the target population of approximately 190 nurses working in the included intensive care units, the minimum required sample size for the current study was calculated to be 128 nurses. This was based on a confidence level of 95%, a margin of error of 5%, and a power of 50%.

### Instruments

A self-administered questionnaire containing five sections was used to gather data. The first section collected data on nurses’ sociodemographic characteristics, including age, gender, marital status, education, and experience.

The second section collected data on nurses’ knowledge of pressure ulcer prevention using the Pressure Ulcer Prevention Knowledge Assessment Instrument (PUPKAI). PUPKAI comprises 34 multiple-choice questions covering several facets of PU detection and prevention, based on current evidence. The instrument contains nine subscale questions generated and adjusted from the Pressure Ulcer Prevention Guideline (PUPG). The items were designed to address several topics, including the definition of pressure ulcers: one item. Another name for pressure ulcers: 1 item. Five items for pressure ulcer causes and risk factors. Three items for the sites of pressure ulcers. Three items represent the signs and symptoms of a pressure ulcer. Four items covered the pressure ulcer assessment. Two items about necessary interventions to avoid pressure ulcers: nutrients and vitamins. Nursing care for pressure ulcers: 14 items, and one item related to pressure ulcer complications (Beeckman et al., 2010; Hamid, 2014; Pieper et al., 1998). The Instrument had a maximum possible score of 34. Knowledge levels were categorized as: satisfactory/proficient (score ≥21, ≥60%), moderate (score 15–20, 44–59%), and poor (score <15, <44%).

The content validity of the PUPKAI was established through evaluation by a panel of three experts in critical care (including two nurse specialists and one intensivist). The experts assessed item relevance using a 4-point scale (1 = not relevant, 4 = highly relevant), yielding a content validity index (CVI) of 0.86, exceeding the recommended threshold of 0.80 for instrument validity. Additionally, the content validity index of the instrument was established in previous studies and confirmed the instrument’s validity (Beeckman et al., 2010; Tulek et al., 2016); moreover, the tool’s reliability was assessed using Cronbach’s α (0.77), which revealed acceptable reliability.

The third section collected data about nurses’ attitudes toward pressure ulcer prevention using the Nurses’ Attitude Scale for Preventing Pressure Ulcers. This scale was adopted to align with our study’s aims (Moore & Price, 2004; Tubaishat, Aljezawi & Al Qadire, 2013) and to measure nurses’ attitudes toward pressure ulcer prevention. The scale consists of 11 items assessing attitudes and beliefs regarding pressure ulcer prevention and care. Each item was rated on a 5-point Likert scale ranging from strongly agree to strongly disagree. The participants rated the degree of attitude (five being strongly disagree, four being disagree, and so on). Nevertheless, the answers to questions 1, 6, 7, and 11 were reverse-scored; for instance, “strongly disagree” was assigned a score of 1, and so on. According to the general guidelines for cut scores on the 5-point Likert scale, a mean attitude score of 1.00 to 2.33 indicates a negative attitude, 2.34 to 3.66 reflects a neutral or mixed attitude, and 3.67 to 5.00 indicates a positive attitude (Jayasinghe et al., 2010).

According to Barakat-Johnson et al. (2018), the score ranged from 11 (most negative attitude) to 55 (most positive attitude). Reliability testing was conducted using Cronbach’s α (0.84). Face and content validity were assessed by a panel of experts (Moore & Price, 2004; Tubaishat, Aljezawi & Al Qadire, 2013). A higher score showed more positive attitudes toward pressure ulcer prevention in patients. The scale is freely and openly available online for use in studies without permission (Agency for Healthcare Research and Quality, 2011). However, permission from the instrument’s author was obtained for its use in this research study.

The fourth section gathered data about nurses’ practices for pressure ulcer prevention in the intensive care unit. The Nurse Pressure Ulcer Practice was adopted from a previous study by Islam (2010); Nuru et al. (2015), and Getie et al. (2020), which consisted of 22 items to evaluate nurses’ practices regarding pressure ulcer prevention in the intensive care unit. Higher ratings indicated better adherence to pressure ulcer prevention measures. Each item was then categorized as ‘done’ and ‘not done. Reliability testing was conducted in the current study using Cronbach’s α (α = −0.82). Both the knowledge and practice tools were re-tested in the current context to confirm their applicability and measurement stability, thereby supporting the robustness of the study’s findings. The validity and reliability of the pressure ulcer prevention practice assessment instrument were assessed in a previous study by Nuru et al. (2015). Before applying the survey instrument, the researchers engaged various expert reviewers, subject matter specialists from Gondar University Hospital, to evaluate and finalize it. For reliability, the study employed Cronbach’s alpha to assess consistency, along with complementary correlation coefficients. A pilot study was conducted to validate the instrument, yielding an overall Cronbach’s alpha of r = 0.76. The instrument is freely and openly available online for use in studies without permission (Nuru et al., 2015).

The fifth section used the Burnout Assessment Tool (BAT 12) as a unique self-report questionnaire to quantify nurses’ burnout (Schaufeli, Desart & De Witte, 2020). The burnout scale comprised 12 items divided equally into four subscales: mental distance, exhaustion, emotional impairment, and cognitive impairment, measured using a 5-point Likert scale. Each subscale measured a diverse feature of burnout twelve items, three of which are on each of the four core burnout syndromes- “At work, I feel mentally exhausted” (exhaustion), “I feel a strong aversion towards my job” (mental distance), “At work, I may overreact unintentionally” (emotional impairment), and “When I am working, I have trouble concentrating” (cognitive impairment)- make up the short version of the burnout assessment scale (Hadžibajramović, Schaufeli & De Witte, 2022). Respondents assessed each issue on a five-point rating system: 1 = never, 2 = seldom, 3 = occasionally, 4 = often, and 5 = always. The BAT-12 provided the total score for each subscale and the overall burnout score.

Based on the findings of a previous study, a score of less than 1.5 indicates a low level of burnout, 1.51 to 2.35 reflects an average level of burnout, 2.36 to 3.17 indicates a high level of burnout, and scores above 3.18 signify a very high level of burnout (Schaufeli & De Witte, 2023). According to Kohnen et al. (2024), the overall burnout scale’s Cronbach’s α was 0.90, whereas in the current study it was 0.88. All efforts were made to ensure accuracy and avoid bias in the measurements of the research instruments.

### Pilot study

Authorization was obtained from the appropriate authorities after formal approval. A pilot study was conducted with 10 nurses who met the study’s inclusion criteria to evaluate the objectivity, transparency, viability, and application of the research instruments. Although no changes were made to the instruments based on the pilot study, the study sample did not include these nurses.

### Data collection procedure

After preparing the data collection instrument, nurses were invited to complete a self-administered paper questionnaire. The data collection process was conducted during both the morning and evening shifts to maximize accessibility and ensure that all eligible nurses had the opportunity to participate at a convenient time. The questionnaires were distributed and collected directly by the researchers, without intermediaries, to maintain control over the data collection process and reduce the risk of selection bias. After completion, nurses placed the questionnaires anonymously into locked collection boxes that were fixed in the intensive care units of the selected hospital to promote confidentiality and reduce social desirability bias. Data collection was carried out over five months, from January 2024 to May 2024.

### Ethical consideration

The study was approved before data collection, and ethical approval was obtained from the Institutional Review Board (IRB) of the Faculty of Nursing at Zagazig University, Egypt (Ethics approval no. ID/ Zu. Nur. REC #:0111 on 1/1/2024). Obtaining official approvals from hospital directors was essential to carry out the current study. Following a thorough explanation of the study’s purpose and goals, the researchers also provided assurances regarding subjects’ voluntary participation, confidentiality, and privacy. Written consent forms were obtained from participants prior to completing the questionnaire.

### Data analysis

After data collection and cleaning, the statistical analysis was conducted using the Statistical Package for the Social Sciences software, SPSS V 21. Descriptive statistics, including mean and standard deviation or frequency and percentage, were used to describe participants’ sociodemographic characteristics and their knowledge, practice, and attitudes regarding pressure ulcer prevention. Moreover, Spearman’s rho was used to assess the correlation among the study’s variables. Consistent with Cohen (1988) guidelines, correlations were interpreted as follows: weak (*r* ≈ 0.10–0.29), moderate (*r* ≈ 0.30–0.49), and strong relationships (*r* ≥ 0.50). The significance of the findings was established at the 5% level. There was no missing data, as all questions and statements in the questionnaire were mandatory.

## Results

This study analyzed responses from 131 nurses—all eligible participants who consented and fully completed the questionnaire—yielding an 83% response rate. The sample size slightly exceeded the minimum requirement, ensuring adequate statistical power to detect meaningful associations in the data.

Table 1 represents the sociodemographic characteristics of the participants. The majority of the 131 participating nurses were women (81.7%, *n* = 107), aged 30–40 years (42.7%, *n* = 56), and married (71.0%, *n* = 93). Most held either a diploma in nursing (40.5%) or a bachelor’s degree (34.4%). Notably, two-thirds (66.4%) had over 10 years of experience, and none reported fewer than 5 years in the field.

**Table 1:** Sociodemographic characteristics of the participant nurses (*N* = 131).

Sociodemographic data		Frequency	Percent
Age	Less than 30 years	41	31.3%
	30 to less than 40 years	56	42.7%
	40 to less than 50 years	30	22.9%
	50 to 60 years	4	3.1%
Gender	Men	24	18.3%
	Women	107	81.7%
Marital status	Married	93	71.0%
	Single	20	15.3%
	Widowed	8	6.1%
	Divorced	10	7.6%
Education	Diploma nurse	53	40.5%
	Technical diploma in nursing	33	25.2%
	Bachelor of Nursing	45	34.4%
Experience	Less than 5 years	0	0.0%
	5 to 10 years	44	33.6%
	More than 10 years	87	66.4%

The subscales in Table 2 measured various aspects of pressure ulcer prevention knowledge, ranging from 0 to 1. The highest mean score was for SS7 (Nutrients and vitamins needed to prevent pressure ulcers) at 0.7 (SD = 0.3) on a scale of 0 to 1, indicating relatively high knowledge in this area. The lowest mean scores were for SS2 (Other nomenclature of pressure ulcer) at 0.3 (SD = 0.4) and SS4 (Pressure Ulcer Sites) at 0.3 (SD = 0.3), suggesting a low level of knowledge in these domains. The total knowledge score across all subscales was 16.1 (SD = 4.6), on a scale of 0 to 34, reflecting moderate knowledge about pressure ulcers. A table of the number of correct responses, mean scores, and standard deviation of the 34 questions of the knowledge assessment scale was included.

**Table 2:** The mean scores of knowledge subscales of the participant nurses.

Knowledge scale	n of correct responses	Mean	SD
*1.SS1: Meaning of pressure ulcers, The definition of pressure ulcer: 1 item*	41	0.3	0.5
*2.SS2: Other nomenclature of pressure ulcer: 1 item*	36	0.3	0.4
3. The main causes of pressure ulcers	73	0.6	0.5
4. The commonest age for developing pressure ulcers	45	0.3	0.5
5. Clients with urinary or fecal incontinence develop pressure ulcers due to	63	0.5	0.5
6. Client under shearing force develops a pressure ulcer due to	71	0.5	0.5
7. Development of pressure ulcers from the wrong method of using a bedpan occurs due to	31	0.2	0.4
*SS3: Causes and risk factors of pressure ulcer: 5 items*		0.4	0.3
8. The point of highest pressure when the client is in the Lateral position	65	0.5	0.5
9. The point of highest pressure in the supine position is	24	0.2	0.4
10. The highest point of pressure in a sitting position	23	0.2	0.4
*SS4: Pressure Ulcer Sites: 3 items*		0.3	0.3
11. The first sign of pressure ulcer development	20	0.2	0.4
12. The following are symptoms of stage III pressure ulcer	85	0.6	0.5
13. The symptom of the unstageable/-Unclassified category of pressure ulcer	89	0.7	0.5
*SS5: Signs and symptoms of pressure ulcer: 3 items*		0.5	0.2
14. The appropriate ways for the assessment of high-risk pressure ulcer development	61	0.5	0.5
15. The appropriate scale for pressure ulcer risk assessment	87	0.7	0.5
16. Frequency of skin assessment	74	0.6	0.5
17. Area for having more attention, while performing a skin assessment	71	0.5	0.5
*SS6: Assessment of Pressure Ulcers: 4 items*		0.6	0.2
18. Vitamins needed to maintain healthy skin	86	0.7	0.5
19. The nutrients needed to prevent pressure ulcers in an elderly patient	92	0.7	0.5
*SS7: Nutrients and vitamins needed to prevent pressure ulcers, 2 items*		0.7	0.3
20. Frequency of cleaning the skin of a client with urinary or fecal incontinence	91	0.7	0.5
21. The agent used for skin cleaning	76	0.6	0.5
22. The frequency of changing the position of a client confined to bed is once	72	0.5	0.5
23. The frequency of changing the position of a client confined to the chair is once	75	0.6	0.5
24. The main purposes of back care	82	0.6	0.5
25. The position used for back care	91	0.7	0.5
26. The agent used during massage to reduce friction is applied	52	0.4	0.5
27. Pressure-relieving devices used to prevent pressure ulcers are	47	0.4	0.5
28. In the supine position, as a supportive device, pillows should be placed,	34	0.3	0.4
29. In the lateral position, pillows should be placed,	38	0.3	0.5
30. An appropriate nursing care for managing mechanical load is,	83	0.6	0.5
31. An appropriate nursing activity to reduce friction	36	0.3	0.4
32. The nursing care for reducing the shearing force	60	0.5	0.5
33. Exercises that prevent pressure ulcers,	51	0.4	0.5
*SS8: Nursing management of pressure ulcers: 14 items*		0.5	0.2
*34.SS9: Complications of pressure ulcers, The most serious complication of pressure ulcers: 1 item*	84	0.6	0.5
**Total knowledge score**		16.1	4.6

Knowledge sub-scales	Mean	SD
SS1: The definition of pressure ulcer: 1 item	.3	.5
SS2: Other nomenclature of pressure ulcer: 1 item	.3	.4
SS3: Causes and risk factors of pressure ulcer: 5 items	.4	.3
SS4: Pressure ulcer sites: 3 items	.3	.3
SS5: Signs and symptoms of pressure ulcer: 3 items	.5	.2
SS6: Assessment of pressure ulcers: 4 items	.6	.2
SS7: Nutrients and vitamins needed to prevent pressure ulcers: 2 items	.7	.3
SS8: Nursing management of pressure ulcers: 14 items	.5	.2
SS9: Complications of pressure ulcers:1 item	.6	.5
**Mean of total knowledge score**	16.1	4.6

**Notes.**

SS: Subscale, SD: standard deviation.

The findings from Table 3 indicate that the majority of participating nurses demonstrated moderately positive attitudes toward pressure ulcer (PU) prevention, though some misconceptions and low prioritization tendencies were observed. Overall, more than half of the nurses (54.8%) strongly disagreed with negative statements about PU prevention, indicating a general awareness of its importance. Most participants recognized the universal risk of PU development, with 30.5% strongly agreeing that all patients are at risk. However, 70.2% strongly disagreed that continuous patient assessment would provide an accurate account of their pressure ulcer risk, and 18.3% strongly agreed that it does. Furthermore, over half of respondents strongly disagreed that PU treatment is a greater priority than prevention (67.2%) and that PU prevention is a low priority in patient care (52.7%), suggesting a preventive care-oriented mindset.

**Table 3:** Mean of attitude scale of the participant nurses toward PU prevention.

Attitude scale	Strongly disagree	Disagree	Neutral	Agree	Strongly agree	Mean	SD
1. All patients are at potential risk of developing pressure ulcers.	51.1%	0.0%	9.2%	9.2%	30.5%	2.7	1.8
2. Pressure ulcer prevention is time-consuming for me to carry out.	31.3%	24.4%	18.3%	22.1%	3.8%	2.4	1.2
3. In my opinion, patients tend not to get as many pressure ulcers nowadays.	58.0%	0.0%	3.1%	15.3%	23.7%	2.5	1.8
4. In my practice, I do not need to concern myself with pressure ulcer prevention.	64.9%	0.8%	6.1%	9.9%	18.3%	2.2	1.7
5. Pressure ulcer treatment is a greater priority than pressure ulcer prevention.	67.2%	4.6%	9.9%	16.0%	2.3%	1.8	1.3
6. Continuous assessment of patients will give an accurate account of their pressure ulcer risk.	70.2%	0.0%	0.0%	11.5%	18.3%	2.1	1.7
7. Most pressure ulcers can be avoided.	52.7%	3.8%	9.2%	11.5%	22.9%	2.5	1.7
8. I am less interested in pressure ulcer prevention than in other aspects of care.	58.0%	0.8%	7.6%	19.1%	14.5%	2.3	1.6
9. My clinical judgment is better than any available pressure ulcer risk assessment tool.	55.0%	10.7%	17.6%	8.4%	8.4%	2.0	1.4
10. In comparison with other areas of care, pressure ulcer prevention is a low priority for me.	52.7%	0.8%	11.5%	5.3%	29.8%	2.6	1.8
11. Pressure ulcer risk assessment should be regularly carried out on all patients during their stay in the hospital.	41.2%	10.7%	12.2%	17.6%	18.3%	2.6	1.6
Mean of the total attitude scale	54.8%	5.1%	9.5%	13.3%	17.3%	2.3	0.5

**Notes.**

SD, standard deviation.

However, moderate levels of neutrality and disagreement in certain areas reflect attitudinal uncertainty. For instance, 18.3% remained neutral about the time burden of prevention activities, and 17.6% were neutral, with 16.8% agreeing that clinical judgment might surpass risk assessment tools, indicating skepticism toward standardized tools. Similarly, only 41.2% strongly disagreed that risk assessments should be routinely performed on all patients during their hospital stay, suggesting partial adherence to preventive protocols.

Overall, while most nurses demonstrated a strongly favorable attitude toward PU prevention and assessment, a minority expressed ambivalence, particularly regarding time constraints and reliance on assessment tools. These findings underscore the need for ongoing education and reinforcement of evidence-based practices for preventing PU.

The highest percentage of nurses in Table 4 reported engaging in practices such as providing vitamins and food (83.2%), documenting all data (82.4%), and using water-filled gloves under the patient’s leg (80.9%), reflecting a commonly observed, improvised practice among nurses in Egyptian hospitals to relieve pressure over bony prominences rather than an evidence- based intervention. In contrast, the lowest percentage of nurses reported performing laboratory tests (3.1%) and assessing and managing pain (5.3%).

**Table 4:** The practice scale scores of the participant nurses (*N* = 131).

Practice scale	Not done		Done	
	**Frequency**	**Percent**	**Frequency**	**Percent**
1. I observe how other nurses assess the risk factors	63	48.1%	68	51.9%
2. I identify common contributing factors to PU	48	36.6%	83	63.4%
3. I do a skin assessment	53	40.5%	78	59.5%
4. I use a risk assessment scale	104	79.4%	27	20.6%
5. I document all data	23	17.6%	108	82.4%
6. I assess and provide management of pain	124	94.7%	7	5.3%
7. I perform skin care as routine work	55	42.0%	76	58.0%
8. I place the pillow under the patient’s leg	89	67.9%	42	32.1%
9. I use water-filled gloves under the patient’s leg	25	19.1%	106	80.9%
10. I use or advise caregivers to use creams or oils	51	38.9%	80	61.1%
11. I pay more attention to pressure points	75	57.3%	56	42.7%
12. I perform lab tests	127	96.9%	4	3.1%
13. I provide vitamins and food	22	16.8%	109	83.2%
14. I monitor a protein and calorie diet	108	82.4%	23	17.6%
15. I avoid dragging	84	64.1%	47	35.9%
16. I always use a special mattress	80	61.1%	51	38.9%
17. I avoid massage	109	83.2%	22	16.8%
18. I avoid using a donut–shaped (ring) cushion	67	51.1%	64	48.9%
19. I turn the patient’s position every two hours.	66	50.4%	65	49.6%
20. I put pillows under the patient’s leg and ankle	63	48.1%	68	51.9%
21. I always attend seminars.	104	79.4%	27	20.6%
22. I give advice to the patient or caregiver.	48	36.6%	83	63.4%
Total practice scale	54.8%		45.2%	

For other practices, the percentages were more evenly distributed, with around 50–60% of nurses reporting that they perform skin assessment, identify contributing factors, and perform routine skin care. Only one-fifth of nurses reported using risk assessment scales, and about half reported turning patients every two hours. In contrast, only 17.6% reported monitoring a protein and calorie-controlled diet. The overall total practice scale score showed that 45.2% of the nurses reported engaging in the assessed pressure ulcer prevention practices, while 54.8% did not.

The mean score for the cognitive impairment subscale in Table 5 was the highest at 3.6 (SD = 0.8), followed by the emotional impairment subscale at 3.6 (SD = 0.9). The mental distance subscale had a mean of 3.4 (SD = 0.8). The exhaustion subscale had the lowest mean of 3.3 (SD = 0.8). The overall mean burnout scale pretest score across all subscales was 3.5 (SD = 0.7), indicating a very high level of burnout among the participant nurses (mean score was greater than 3.18).

**Table 5:** Mean burnout scale of the participant nurses on a 1–5 scale (*N* = 131).

**Burnout Scale**	**Mean**	**SD**
1. I feel mentally exhausted	3.3	1.2
2. At the end of the day, I find it hard to recover my energy	3.3	1.2
3. I feel physically exhausted	3.5	1.2
*Exhaustion subscale*	3.3	0.8
4. I struggle to find any enthusiasm for my work	3.3	1.1
5. I feel a strong aversion towards my job	3.2	1.2
6. I am cynical about what my work means to others	3.7	1.1
*Mental distance subscale*	3.4	0.8
7. I have trouble staying focused	3.6	1.0
8. I have trouble concentrating	3.7	1.0
9. I make mistakes because I have my mind on other things	3.5	1.2
*Cognitive impairment subscale*	3.6	0.8
10. I feel unable to control my emotions	3.7	0.9
11. I do not recognize myself in the way I react emotionally	3.5	1.1
12. I may overreact unintentionally	3.6	0.9
*Emotional Impairment Subscale*	3.6	0.9
Mean-Score of the Burnout Scale	3.5	0.7

Participants’ knowledge and practice in Table 6 showed a moderate positive correlation (*r* = 0.353, *p* < 0.001), suggesting that higher knowledge scores were associated with better adherence to prevention practices. Knowledge and Burnout exhibited a weak-to-moderate positive correlation (*r* = 0.295, *p* = 0.001), suggesting a slight tendency for nurses with higher knowledge to report higher burnout. However, nurses’ attitudes and practices showed a weak positive correlation (*r* = 0.194, *p* = 0.026), suggesting that more favorable attitudes were only minimally associated with improved practices. All other correlations were negligible and statistically non-significant (*p* > 0.05), including Attitude and Burnout (*r* = −0.076, *p* = 0.390), and Practice and Burnout (*r* = 0.146, *p* = 0.097). Consistent with Cohen (1988) guidelines, correlations were interpreted as follows: weak (*r* ≈ 0.10–0.29), moderate (*r* ≈ 0.30–0.49), and strong (*r* ≥ 0.50).

**Table 6:** Correlation among nurses’ knowledge, attitudes, and practices of pressure ulcer prevention and their burnout level (*N* = 131).

Correlations			Knowledge score	Attitude score	Practice score	Burnout scale
Spearman’s rho	Knowledge acore	Correlation coefficient	>0.999	−0.008	0.353	0.295
		Sig. (2-tailed)		0.931	<0.001^**^	<0.001^**^
	Attitude score	Correlation coefficient	−0.008	>0.999	0.194	−0.076
		Sig. (2-tailed)	0.931		0.026^*^	390
	Practice score	Correlation coefficient	0.353	0.194	>0.999	0.146
		Sig. (2-tailed)	<0.001^**^	0.026^*^		0.097

**Notes.**

** Correlation is significant at *p* < .010 level (2-tailed).

* Correlation is significant at *p* < .050 level (2-tailed).

## Discussion

The current study aimed to explore nurses’ knowledge, attitudes, and practices regarding pressure ulcer prevention in intensive care units. Additionally, it sought to identify any correlation between these variables and nurse burnout. The major findings of this study revealed that nurses demonstrated a moderate understanding of various aspects of pressure ulcer prevention, particularly the vitamins and nutrients necessary for preventing and mitigating the consequences of pressure ulcers. However, there were areas, such as the anatomy and lexicon of pressure ulcers, where their expertise was lacking. This suggests that although the nurses were not fully equipped to implement pressure ulcer prevention, their knowledge was moderate and could be significantly improved.

Research on nurses’ knowledge of pressure ulcer prevention presents mixed findings. While some studies report inadequate knowledge among nurses (Alhawsah et al., 2025; Ebi, Hirko & Mijena, 2019; Gedamu et al., 2021; Wu et al., 2022), others indicate more positive results (Abebe & Daniel, 2015; Inayat et al., 2025). Despite knowledge gaps, nurses generally demonstrated positive attitudes toward pressure ulcer prevention (Abebe & Daniel, 2015; Inayat et al., 2025). Common barriers to prevention included a shortage of pressure-relieving devices, understaffing, and a lack of training (Ebi, Hirko & Mijena, 2019). These findings highlight the need for regular training and education to enhance nurses’ knowledge and improve pressure ulcer prevention practices.

These findings align with an earlier Chinese study that highlighted the need for more effective training in pre-registration nursing schools to administer PUs prevention programs in intensive care units (Li et al., 2022b). Another study also found that ICU nurses had limited comprehension of PU prevention (Hu, Sae-Sia & Kitrungrote, 2021). Moreover, a meta-analysis found that understanding pressure injury prevention among nurses and nursing students is unsatisfactory, underscoring the urgent need for continuous learning (Tian et al., 2023).

The outcomes of the present study indicated that ICU nurses generally had a moderately positive attitude toward preventing pressure ulcers. The individuals acknowledged the importance of evaluating and preventing pressure ulcer risk, and the majority believed that, with adequate care and attention, most pressure ulcers can be prevented. A positive mindset is essential for implementing effective pressure ulcer prevention techniques in the ICU setting. Nurses who prioritized preventing pressure ulcers and understood their clinical significance were more likely to implement evidence-based prevention strategies. These strategies—emphasized in the pressure ulcer prevention guidelines by the National Pressure Ulcer Advisory Panel (NPUAP), the European Pressure Ulcer Advisory Panel (EPUAP), and the Pan Pacific Pressure Injury Alliance (PPPIA)—include routine risk assessment, skin inspection, proper support surfaces, and regular repositioning (Kottner et al., 2019; Kottner et al., 2020). A study by Wan et al. (2023) examined and supported this bundle of preventive interventions in clinical settings, exploring both the experiences of evidence-based pressure injury practices and the barriers and facilitators to guideline implementation.

Previous research indicated that nurses generally have positive attitudes toward pressure ulcer prevention. Studies across different healthcare settings, including long-term care facilities in Korea and hospitals in Cyprus and Finland, consistently report favorable attitudes among nursing staff (Charalambous et al., 2019; Kim & Lee, 2019; Li et al., 2022a; Parisod et al., 2022; Zhang et al., 2021). However, translating these positive attitudes into effective prevention practices remains challenging (Avsar et al., 2019). Likewise, our findings are consistent with the most recent study, which found that nurses lacked sufficient understanding but generally had positive perspectives and attitudes toward PU prevention (Li et al., 2022a). This highlights the significance of providing ongoing education and training on pressure ulcer prevention in healthcare settings to boost the quality of service (Liang et al., 2024).

In this study, we found that nurses’ engagement in procedures to prevent pressure ulcers was inconsistent. Despite this, most nurses reported performing tasks such as keeping records, documenting, and administering sustenance. Risk assessment tools, pain management, calorie and protein dietary monitoring, and frequent repositioning were among the essential preventive measures that many nurses did not implement. These findings are consistent with Haavisto et al. (2021), who reported that although prevention practices were generally followed “quite frequently”, notable gaps in adherence were observed, particularly in nutrition, and that overall agreement with guideline-based practices remained inadequate. Better pressure ulcer prevention practices were associated with higher levels of knowledge among intensive care unit nurses, suggesting that their attitudes and knowledge play a significant role in shaping their clinical practices, as supported by previous studies (Alshahrani et al., 2023; Asiri et al., 2025). The intensive care unit nursing staff may benefit from enhanced education and the implementation of comprehensive guidelines and ICU-specific pressure ulcer prevention protocols to promote consistent preventive practices in high-risk critical care settings.

The current investigation also identified several obstacles to successful pressure ulcer prevention, including the belief among some nurses that it is a time-consuming process (25.9%) and that one’s clinical judgment is superior to formal risk assessment techniques (16.8%). We could attribute these views to a scarcity of personnel and inadequate resources. These attitudes may impede the consistent implementation of pressure ulcer prevention methods and the use of available tools and guidance. To enhance pressure ulcer prevention in ICUs, it is crucial to overcome these attitudinal obstacles by implementing targeted education and training and fostering a culture that prioritizes pressure ulcer prevention as an integral component of patient care. Cultivating a resilient, optimistic, and proactive mindset among ICU nurses can increase the probability of effectively preventing pressure ulcers and enhancing patient outcomes.

The findings of this study correspond with those of a previous Spanish study, which similarly highlighted that a lack of staff and resources impedes the successful adoption of care techniques to reduce pressure ulcers (Acosta-Hernández et al., 2023). Additionally, previous systematic reviews found that nurses’ knowledge and attitudes had a beneficial impact on their practice in preventing PUs; thus, it is vital to provide PUs-related training to improve nurses’ knowledge and attitudes (Fang et al., 2025; Saleh et al., 2026). In the same vein, our findings confirmed those of an earlier study, which found that intensive care unit nurses have favorable views about PU prevention but limited knowledge. Policies and procedures should be established to enhance ICU nurses’ knowledge and eliminate barriers to implementing optimal PU prevention practices (Aydogan & Caliskan, 2019).

A recent study in Iran highlighted the importance of addressing key obstacles that hinder ICU nurses’ efforts to prevent pressure ulcers. These barriers include heavy workloads, insufficient nurse staffing, and inadequate prevention guidelines. The study suggests that nurse managers and policymakers should prioritize eliminating these barriers to improve PU prevention practice among ICU nurses (Ghazanfari et al., 2022). Furthermore, our research concurs with previous studies that have demonstrated the positive impact of the training program on nurses’ knowledge, attitudes, and confidence in pressure injury care (Chuang et al., 2022).

Recent studies have consistently shown positive correlations between nurses’ knowledge and attitudes regarding pressure ulcer prevention (Bakar, Abdullah & Sohor, 2024; Charalambous et al., 2019; Grešš Halász et al., 2021; Khojastehfar, Najafi Ghezeljeh & Haghani, 2020). Oppositely, Ghazanfari et al. (2022) found a negative correlation between knowledge and attitude.

Our results align with a Saudi multicenter study that found that nurses’ training and instruction are crucial for enhancing PU prevention strategies and decreasing PU occurrence, particularly in intensive care units (Alshahrani et al., 2023). Specifically, both studies demonstrate that ICU nurses’ baseline knowledge and attitudes regarding pressure ulcer prevention are suboptimal, and that higher knowledge levels are positively associated with more favorable attitudes and better preventive practices. Alshahrani et al. (2023) further demonstrated that a tailored, evidence-based educational intervention significantly improved nurses’ knowledge and attitudes, supporting our interpretation that structured training and ongoing instruction are crucial strategies for strengthening ICU nurses’ pressure ulcer prevention practices and potentially reducing the incidence of pressure ulcers. Additionally, many studies highlighted the importance of training in PU preventive knowledge (Hu, Sae-Sia & Kitrungrote, 2021; Song et al., 2024; Yazıcıet al., 2022).

The findings of our study reveal that ICU nurses exhibit very high levels of burnout, particularly in cognitive and emotional functioning. This suggests that the nurses were experiencing mental exhaustion, impaired focus, and emotional anguish, which could negatively affect their capacity to deliver exceptional patient care and their overall state of well-being. The results emphasize the importance of addressing burnout and establishing efficient ways to meet the psychological and emotional needs of nursing personnel in the ICU environment. Previous research supported our study’s findings, which revealed that nurses employed in intensive care units in Saudi Arabia experienced varying degrees of burnout, ranging from moderate to high levels (Alzailai, Barriball & Xyrichis, 2021; Shahin, 2025). An Indian study noted a high prevalence of burnout among intensive care nurses (Kumar et al., 2021).

Our findings endorse a prior study demonstrating a statistically significant correlation between burnout and nurses’ physical and mental well-being. In addition, burnout diminishes the standard of care and increases the likelihood of errors in clinical practice, resulting in negative consequences for the nursing profession, such as termination or voluntary departure (Ślusarz et al., 2022).

The current study reported a weak positive association between knowledge and burnout, suggesting that greater knowledge about PU may also be associated with higher levels of burnout among nurses. At first glance, one might assume that nurses with greater knowledge about PU would experience less burnout, empowered by their expertise; however, our findings suggest a more nuanced reality—that deeper understanding may paradoxically become an emotional and professional burden. In resource-limited settings common to many hospitals, these nurses experience moral injury when their knowledge outstrips their ability to act, knowing that evidence supports interventions such as two-hour turn schedules or advanced wound care supplies but lacking the staff or budget to implement them (Qaddumi & Khawaldeh, 2014). This tension between knowing what should be done and being unable to do it ideally may help explain why more knowledgeable participants reported greater exhaustion, as their heightened awareness of prevention obligations increases cognitive and emotional burnout, ultimately leading to greater physical exhaustion in their efforts to achieve optimal patient care in preventing PUs.

This highlights the need for a multifaceted approach to supporting ICU nurses, one that addresses not only their knowledge and practices but also their overall well-being and burnout levels. Strategies to enhance both the clinical competence and the psychological and emotional resilience of the nursing staff may be crucial in improving pressure ulcer prevention and overall patient care in the ICU setting.

The findings of this study were supported by previous research, which recommended implementing resilience programs to prevent burnout and improve the ability to deliver high-quality healthcare (Castillo-González et al., 2023). A recent study found that psychological intervention can positively impact empathy fatigue, empathy satisfaction, and burnout among nursing staff. Furthermore, it has been shown to enhance their overall quality of life (Chen et al., 2022). Additional interventions should be developed to assist nurses in effectively managing distressing emotions, bereavement, and grief, while also implementing practical interventions for patients (Yıldız, 2023).

## Conclusions

According to the current study, intensive care unit nurses demonstrated moderate knowledge and a moderately positive attitude toward preventing pressure ulcers. However, they also recognized several potential obstacles to effective pressure ulcer prevention, including the belief that it takes too much time and that nurses’ clinical judgment is superior to official PU risk assessment tools. The ICU nurses experienced a very high level of burnout, with the highest levels observed in cognitive and emotional impairment. A moderate positive correlation was found between participants’ knowledge and practice, indicating that higher knowledge scores were associated with greater adherence to prevention measures. Moreover, nurses’ attitudes and practices showed only a weak positive correlation, suggesting that more favorable attitudes were only minimally associated with improved practices. Intensive care nurses’ knowledge and attitudes regarding pressure ulcer prevention are essential influences on their clinical practices. However, the weak positive association between knowledge and burnout suggests that greater knowledge about PU may also be associated with higher levels of burnout among nurses. This underscores the urgency and importance of a multifaceted approach to supporting ICU nurses, one that considers their knowledge and practices, as well as their overall well-being and burnout levels.

### Recommendations

Nurses’ knowledge and attitudes regarding pressure ulcers significantly influence their practices for preventing and managing PUs in patients in intensive care units. Therefore, implementing targeted educational programs may enhance nursing practices in this area. Interventions must be made to assist nurses in efficiently managing distressing emotions, burnout, bereavement, and grief while also carrying out practical interventions with PU patients. It is crucial to address nurses’ specific learning needs and foster a positive outlook on this critical aspect of healthcare by creating and utilizing coping mechanisms to support medical personnel’s health and well-being in high-stress situations. In addition, it is imperative to evaluate variables that extend beyond the individual, including nursing leadership, hospital climate, and cultural and contextual influences.

### Limitations

This research had some limitations. For instance, it relied on self-reported knowledge, attitudes, and practice questionnaires on pressure ulcer prevention without direct assessment or observation of nurses, which may have introduced high subjectivity into the findings. The questionnaires were distributed and collected directly by the researchers during routine shifts, which may have influenced response rates and participation; for example, nurses who were busier or absent during the data collection period may have been less likely to respond. Additionally, data were collected from single-region hospitals in Egypt, which may limit the generalizability of the findings.

## Supplemental Information

10.7717/peerj.21250/supp-1 Supplemental Information 1 Dataset

10.7717/peerj.21250/supp-2 Supplemental Information 2 SPSS data

10.7717/peerj.21250/supp-3 Supplemental Information 3 Codebook

10.7717/peerj.21250/supp-4 Supplemental Information 4 STROBE checklist

10.7717/peerj.21250/supp-5 Supplemental Information 5 Questionnaire

## Additional Information and Declarations

### Data Availability

The following information was supplied regarding data availability: The raw data is available in the Supplemental Files.
