# Supplementary material for: Nurses’ knowledge, attitudes, and practices in pressure ulcer prevention in intensive care units: associations with burnout
Source: PeerJ. 2026 May 14;14:e21250. doi: 10.7717/peerj.21250 (PMC13180348; doi:10.7717/peerj.21250)
Supplement: Supplemental Information 3 [file peerj-14-21250-s003.docx]

**Codebook**

| **Age** | | | | |
| --- | --- | --- | --- | --- |
|  | | Value | Count | Percent |
| Standard Attributes | Position | 1 |  |  |
|  | Label | Age |  |  |
|  | Type | Numeric |  |  |
|  | Format | F8 |  |  |
|  | Measurement | Nominal |  |  |
|  | Role | Input |  |  |
| Valid Values | 1 | less than 20 years | 0 | 0.0% |
|  | 2 | 20 t0 less than 30 years | 41 | 31.3% |
|  | 3 | 30 t0 less than 40 years | 56 | 42.7% |
|  | 4 | 40 to less than 50 years | 30 | 22.9% |
|  | 5 | 50 t0 60 years | 4 | 3.1% |

| **Gender** | | | | |
| --- | --- | --- | --- | --- |
|  | | Value | Count | Percent |
| Standard Attributes | Position | 2 |  |  |
|  | Label | Gender |  |  |
|  | Type | Numeric |  |  |
|  | Format | F8 |  |  |
|  | Measurement | Nominal |  |  |
|  | Role | Input |  |  |
| Valid Values | 1 | male | 24 | 18.3% |
|  | 2 | female | 107 | 81.7% |

| **maritalstatus** | | | | |
| --- | --- | --- | --- | --- |
|  | | Value | Count | Percent |
| Standard Attributes | Position | 3 |  |  |
|  | Label | Marital status |  |  |
|  | Type | Numeric |  |  |
|  | Format | F8 |  |  |
|  | Measurement | Nominal |  |  |
|  | Role | Input |  |  |
| Valid Values | 1 | married | 93 | 71.0% |
|  | 2 | single | 20 | 15.3% |
|  | 3 | widower | 8 | 6.1% |
|  | 4 | divorced | 10 | 7.6% |

| **education** | | | | |
| --- | --- | --- | --- | --- |
|  | | Value | Count | Percent |
| Standard Attributes | Position | 4 |  |  |
|  | Label | Education |  |  |
|  | Type | Numeric |  |  |
|  | Format | F8 |  |  |
|  | Measurement | Nominal |  |  |
|  | Role | Input |  |  |
| Valid Values | 1 | diploma nurse | 53 | 40.5% |
|  | 2 | technical diploma nursing | 33 | 25.2% |
|  | 3 | technical institute health | 0 | 0.0% |
|  | 4 | Bachelor of nursing | 45 | 34.4% |

| **experienceyears** | | | | |
| --- | --- | --- | --- | --- |
|  | | Value | Count | Percent |
| Standard Attributes | Position | 5 |  |  |
|  | Label | Experienceyears |  |  |
|  | Type | Numeric |  |  |
|  | Format | F8 |  |  |
|  | Measurement | Nominal |  |  |
|  | Role | Input |  |  |
| Valid Values | 1 | less than one year | 0 | 0.0% |
|  | 2 | 1 to 5 years | 0 | 0.0% |
|  | 3 | 5 to 10 years | 44 | 33.6% |
|  | 4 | more than 10 years | 87 | 66.4% |

| **VAR00006** | | |
| --- | --- | --- |
|  | | Value |
| Standard Attributes | Position | 6 |
|  | Label | //////////////////////////////////////////////////////////////////////////////////////////////////////////////////////////////////////////////////////// |
|  | Type | Numeric |
|  | Format | F8.2 |
|  | Measurement | Scale |
|  | Role | Input |
| N | Valid | 0 |
|  | Missing | 131 |
| Central Tendency and Dispersion | Mean | . |
|  | Standard Deviation | . |
|  | Percentile 25 | . |
|  | Percentile 50 | . |
|  | Percentile 75 | . |

| **k.1.pre** | | | | |
| --- | --- | --- | --- | --- |
|  | | Value | Count | Percent |
| Standard Attributes | Position | 7 |  |  |
|  | Label | SS1: Meaning of pressure sores, The definition of pressure sore: 1 item |  |  |
|  | Type | Numeric |  |  |
|  | Format | F8 |  |  |
|  | Measurement | Scale |  |  |
|  | Role | Input |  |  |
| N | Valid | 131 |  |  |
|  | Missing | 0 |  |  |
| Central Tendency and Dispersion | Mean | .31 |  |  |
|  | Standard Deviation | .465 |  |  |
|  | Percentile 25 | .00 |  |  |
|  | Percentile 50 | .00 |  |  |
|  | Percentile 75 | 1.00 |  |  |
| Labeled Values | 0 | not correct answer | 90 | 68.7% |
|  | 1 | correct answer | 41 | 31.3% |

| **k.2.pre** | | | | |
| --- | --- | --- | --- | --- |
|  | | Value | Count | Percent |
| Standard Attributes | Position | 8 |  |  |
|  | Label | SS2: Other nomenclature of pressure ulcer: 1 item |  |  |
|  | Type | Numeric |  |  |
|  | Format | F8 |  |  |
|  | Measurement | Scale |  |  |
|  | Role | Input |  |  |
| N | Valid | 131 |  |  |
|  | Missing | 0 |  |  |
| Central Tendency and Dispersion | Mean | .27 |  |  |
|  | Standard Deviation | .448 |  |  |
|  | Percentile 25 | .00 |  |  |
|  | Percentile 50 | .00 |  |  |
|  | Percentile 75 | 1.00 |  |  |
| Labeled Values | 0 | not correct answer | 95 | 72.5% |
|  | 1 | correct answer | 36 | 27.5% |

| **k.3.pre** | | | | |
| --- | --- | --- | --- | --- |
|  | | Value | Count | Percent |
| Standard Attributes | Position | 9 |  |  |
|  | Label | The main causes of pressure ulceri, SS3: Causes and risk factors of pressure sore: 5 items |  |  |
|  | Type | Numeric |  |  |
|  | Format | F8 |  |  |
|  | Measurement | Scale |  |  |
|  | Role | Input |  |  |
| N | Valid | 131 |  |  |
|  | Missing | 0 |  |  |
| Central Tendency and Dispersion | Mean | .56 |  |  |
|  | Standard Deviation | .499 |  |  |
|  | Percentile 25 | .00 |  |  |
|  | Percentile 50 | 1.00 |  |  |
|  | Percentile 75 | 1.00 |  |  |
| Labeled Values | 0 | not correct answer | 58 | 44.3% |
|  | 1 | correct answer | 73 | 55.7% |

| **k.4.pre** | | | | |
| --- | --- | --- | --- | --- |
|  | | Value | Count | Percent |
| Standard Attributes | Position | 10 |  |  |
|  | Label | The commonest age for developing pressure sore |  |  |
|  | Type | Numeric |  |  |
|  | Format | F8 |  |  |
|  | Measurement | Scale |  |  |
|  | Role | Input |  |  |
| N | Valid | 131 |  |  |
|  | Missing | 0 |  |  |
| Central Tendency and Dispersion | Mean | .34 |  |  |
|  | Standard Deviation | .477 |  |  |
|  | Percentile 25 | .00 |  |  |
|  | Percentile 50 | .00 |  |  |
|  | Percentile 75 | 1.00 |  |  |
| Labeled Values | 0 | not correct answer | 86 | 65.6% |
|  | 1 | correct answer | 45 | 34.4% |

| **k.5.pre** | | | | |
| --- | --- | --- | --- | --- |
|  | | Value | Count | Percent |
| Standard Attributes | Position | 11 |  |  |
|  | Label | Clients with urinary or fecal incontinence develop pressure sore due to |  |  |
|  | Type | Numeric |  |  |
|  | Format | F8 |  |  |
|  | Measurement | Scale |  |  |
|  | Role | Input |  |  |
| N | Valid | 131 |  |  |
|  | Missing | 0 |  |  |
| Central Tendency and Dispersion | Mean | .48 |  |  |
|  | Standard Deviation | .502 |  |  |
|  | Percentile 25 | .00 |  |  |
|  | Percentile 50 | .00 |  |  |
|  | Percentile 75 | 1.00 |  |  |
| Labeled Values | 0 | not correct answer | 68 | 51.9% |
|  | 1 | correct answer | 63 | 48.1% |

| **k.6.pre** | | | | |
| --- | --- | --- | --- | --- |
|  | | Value | Count | Percent |
| Standard Attributes | Position | 12 |  |  |
|  | Label | Client under shearing force develop pressure sore due to |  |  |
|  | Type | Numeric |  |  |
|  | Format | F8 |  |  |
|  | Measurement | Scale |  |  |
|  | Role | Input |  |  |
| N | Valid | 131 |  |  |
|  | Missing | 0 |  |  |
| Central Tendency and Dispersion | Mean | .54 |  |  |
|  | Standard Deviation | .500 |  |  |
|  | Percentile 25 | .00 |  |  |
|  | Percentile 50 | 1.00 |  |  |
|  | Percentile 75 | 1.00 |  |  |
| Labeled Values | 0 | not correct answer | 60 | 45.8% |
|  | 1 | correct answer | 71 | 54.2% |

| **k.7.pre** | | | | |
| --- | --- | --- | --- | --- |
|  | | Value | Count | Percent |
| Standard Attributes | Position | 13 |  |  |
|  | Label | Development of pressure sores from wrong method of using bed pan occurs due to |  |  |
|  | Type | Numeric |  |  |
|  | Format | F8 |  |  |
|  | Measurement | Scale |  |  |
|  | Role | Input |  |  |
| N | Valid | 131 |  |  |
|  | Missing | 0 |  |  |
| Central Tendency and Dispersion | Mean | .24 |  |  |
|  | Standard Deviation | .427 |  |  |
|  | Percentile 25 | .00 |  |  |
|  | Percentile 50 | .00 |  |  |
|  | Percentile 75 | .00 |  |  |
| Labeled Values | 0 | not correct answer | 100 | 76.3% |
|  | 1 | correct answer | 31 | 23.7% |

| **SS3.pre** | | |
| --- | --- | --- |
|  | | Value |
| Standard Attributes | Position | 14 |
|  | Label | SS3: Causes and risk factors of pressure sore: 5 items |
|  | Type | Numeric |
|  | Format | F8.2 |
|  | Measurement | Scale |
|  | Role | Input |
| N | Valid | 131 |
|  | Missing | 0 |
| Central Tendency and Dispersion | Mean | .4321 |
|  | Standard Deviation | .27154 |
|  | Percentile 25 | .2000 |
|  | Percentile 50 | .4000 |
|  | Percentile 75 | .6000 |

| **k.8.pre** | | | | |
| --- | --- | --- | --- | --- |
|  | | Value | Count | Percent |
| Standard Attributes | Position | 15 |  |  |
|  | Label | The point of highest pressure when the client is in Lateral position, SS4: Sites: 3 items |  |  |
|  | Type | Numeric |  |  |
|  | Format | F8 |  |  |
|  | Measurement | Scale |  |  |
|  | Role | Input |  |  |
| N | Valid | 131 |  |  |
|  | Missing | 0 |  |  |
| Central Tendency and Dispersion | Mean | .50 |  |  |
|  | Standard Deviation | .502 |  |  |
|  | Percentile 25 | .00 |  |  |
|  | Percentile 50 | .00 |  |  |
|  | Percentile 75 | 1.00 |  |  |
| Labeled Values | 0 | not correct answer | 66 | 50.4% |
|  | 1 | correct answer | 65 | 49.6% |

| **k.9.pre** | | | | |
| --- | --- | --- | --- | --- |
|  | | Value | Count | Percent |
| Standard Attributes | Position | 16 |  |  |
|  | Label | The point of highest pressure in supine position is |  |  |
|  | Type | Numeric |  |  |
|  | Format | F8 |  |  |
|  | Measurement | Scale |  |  |
|  | Role | Input |  |  |
| N | Valid | 131 |  |  |
|  | Missing | 0 |  |  |
| Central Tendency and Dispersion | Mean | .18 |  |  |
|  | Standard Deviation | .388 |  |  |
|  | Percentile 25 | .00 |  |  |
|  | Percentile 50 | .00 |  |  |
|  | Percentile 75 | .00 |  |  |
| Labeled Values | 0 | not correct answer | 107 | 81.7% |
|  | 1 | correct answer | 24 | 18.3% |

| **k.10.pre** | | | | |
| --- | --- | --- | --- | --- |
|  | | Value | Count | Percent |
| Standard Attributes | Position | 17 |  |  |
|  | Label | The point of highest pressure In a sitting position |  |  |
|  | Type | Numeric |  |  |
|  | Format | F8 |  |  |
|  | Measurement | Scale |  |  |
|  | Role | Input |  |  |
| N | Valid | 131 |  |  |
|  | Missing | 0 |  |  |
| Central Tendency and Dispersion | Mean | .18 |  |  |
|  | Standard Deviation | .382 |  |  |
|  | Percentile 25 | .00 |  |  |
|  | Percentile 50 | .00 |  |  |
|  | Percentile 75 | .00 |  |  |
| Labeled Values | 0 | not correct answer | 108 | 82.4% |
|  | 1 | correct answer | 23 | 17.6% |

| **SS4.pre** | | |
| --- | --- | --- |
|  | | Value |
| Standard Attributes | Position | 18 |
|  | Label | SS4: Pressure Ulcer Sites: 3 items |
|  | Type | Numeric |
|  | Format | F8.2 |
|  | Measurement | Scale |
|  | Role | Input |
| N | Valid | 131 |
|  | Missing | 0 |
| Central Tendency and Dispersion | Mean | .2850 |
|  | Standard Deviation | .29271 |
|  | Percentile 25 | .0000 |
|  | Percentile 50 | .3333 |
|  | Percentile 75 | .3333 |

| **k.11.pre** | | | | |
| --- | --- | --- | --- | --- |
|  | | Value | Count | Percent |
| Standard Attributes | Position | 19 |  |  |
|  | Label | The first sign for pressure ulcer development, SS5: Signs and symptoms of pressure sore: 3 items |  |  |
|  | Type | Numeric |  |  |
|  | Format | F8 |  |  |
|  | Measurement | Scale |  |  |
|  | Role | Input |  |  |
| N | Valid | 131 |  |  |
|  | Missing | 0 |  |  |
| Central Tendency and Dispersion | Mean | .15 |  |  |
|  | Standard Deviation | .361 |  |  |
|  | Percentile 25 | .00 |  |  |
|  | Percentile 50 | .00 |  |  |
|  | Percentile 75 | .00 |  |  |
| Labeled Values | 0 | not correct answer | 111 | 84.7% |
|  | 1 | correct answer | 20 | 15.3% |

| **k.12.pre** | | | | |
| --- | --- | --- | --- | --- |
|  | | Value | Count | Percent |
| Standard Attributes | Position | 20 |  |  |
|  | Label | The following are symptom of stage III pressure ulcer |  |  |
|  | Type | Numeric |  |  |
|  | Format | F8 |  |  |
|  | Measurement | Scale |  |  |
|  | Role | Input |  |  |
| N | Valid | 131 |  |  |
|  | Missing | 0 |  |  |
| Central Tendency and Dispersion | Mean | .65 |  |  |
|  | Standard Deviation | .479 |  |  |
|  | Percentile 25 | .00 |  |  |
|  | Percentile 50 | 1.00 |  |  |
|  | Percentile 75 | 1.00 |  |  |
| Labeled Values | 0 | not correct answer | 46 | 35.1% |
|  | 1 | correct answer | 85 | 64.9% |

| **k.13.pre** | | | | |
| --- | --- | --- | --- | --- |
|  | | Value | Count | Percent |
| Standard Attributes | Position | 21 |  |  |
|  | Label | The symptom of unstageable/ Unclassified category of pressure ulcer |  |  |
|  | Type | Numeric |  |  |
|  | Format | F8 |  |  |
|  | Measurement | Scale |  |  |
|  | Role | Input |  |  |
| N | Valid | 131 |  |  |
|  | Missing | 0 |  |  |
| Central Tendency and Dispersion | Mean | .68 |  |  |
|  | Standard Deviation | .469 |  |  |
|  | Percentile 25 | .00 |  |  |
|  | Percentile 50 | 1.00 |  |  |
|  | Percentile 75 | 1.00 |  |  |
| Labeled Values | 0 | not correct answer | 42 | 32.1% |
|  | 1 | correct answer | 89 | 67.9% |

| **SS5.pre** | | |
| --- | --- | --- |
|  | | Value |
| Standard Attributes | Position | 22 |
|  | Label | SS5: Signs and symptoms of pressure sore: 3 items |
|  | Type | Numeric |
|  | Format | F8.2 |
|  | Measurement | Scale |
|  | Role | Input |
| N | Valid | 131 |
|  | Missing | 0 |
| Central Tendency and Dispersion | Mean | .4936 |
|  | Standard Deviation | .24582 |
|  | Percentile 25 | .3333 |
|  | Percentile 50 | .6667 |
|  | Percentile 75 | .6667 |

| **k.14.pre** | | | | |
| --- | --- | --- | --- | --- |
|  | | Value | Count | Percent |
| Standard Attributes | Position | 23 |  |  |
|  | Label | The appropriate ways for assessment for high risk pressure ulcer development, SS6: Assessment of Pressure sores: 4 items |  |  |
|  | Type | Numeric |  |  |
|  | Format | F8 |  |  |
|  | Measurement | Scale |  |  |
|  | Role | Input |  |  |
| N | Valid | 131 |  |  |
|  | Missing | 0 |  |  |
| Central Tendency and Dispersion | Mean | .47 |  |  |
|  | Standard Deviation | .501 |  |  |
|  | Percentile 25 | .00 |  |  |
|  | Percentile 50 | .00 |  |  |
|  | Percentile 75 | 1.00 |  |  |
| Labeled Values | 0 | not correct answer | 70 | 53.4% |
|  | 1 | correct nswer | 61 | 46.6% |

| **k.15.pre** | | | | |
| --- | --- | --- | --- | --- |
|  | | Value | Count | Percent |
| Standard Attributes | Position | 24 |  |  |
|  | Label | The appropriate scale for pressure ulcer risk assessment |  |  |
|  | Type | Numeric |  |  |
|  | Format | F8 |  |  |
|  | Measurement | Scale |  |  |
|  | Role | Input |  |  |
| N | Valid | 131 |  |  |
|  | Missing | 0 |  |  |
| Central Tendency and Dispersion | Mean | .66 |  |  |
|  | Standard Deviation | .474 |  |  |
|  | Percentile 25 | .00 |  |  |
|  | Percentile 50 | 1.00 |  |  |
|  | Percentile 75 | 1.00 |  |  |
| Labeled Values | 0 | not correct answer | 44 | 33.6% |
|  | 1 | correct answer | 87 | 66.4% |

| **k.16.pre** | | | | |
| --- | --- | --- | --- | --- |
|  | | Value | Count | Percent |
| Standard Attributes | Position | 25 |  |  |
|  | Label | Frequency of skin assessment |  |  |
|  | Type | Numeric |  |  |
|  | Format | F8 |  |  |
|  | Measurement | Scale |  |  |
|  | Role | Input |  |  |
| N | Valid | 131 |  |  |
|  | Missing | 0 |  |  |
| Central Tendency and Dispersion | Mean | .56 |  |  |
|  | Standard Deviation | .498 |  |  |
|  | Percentile 25 | .00 |  |  |
|  | Percentile 50 | 1.00 |  |  |
|  | Percentile 75 | 1.00 |  |  |
| Labeled Values | 0 | not correct answer | 57 | 43.5% |
|  | 1 | correct answer | 74 | 56.5% |

| **k.17.pre** | | | | |
| --- | --- | --- | --- | --- |
|  | | Value | Count | Percent |
| Standard Attributes | Position | 26 |  |  |
|  | Label | Area for having more attention, while performing skin assessment |  |  |
|  | Type | Numeric |  |  |
|  | Format | F8 |  |  |
|  | Measurement | Scale |  |  |
|  | Role | Input |  |  |
| N | Valid | 131 |  |  |
|  | Missing | 0 |  |  |
| Central Tendency and Dispersion | Mean | .54 |  |  |
|  | Standard Deviation | .500 |  |  |
|  | Percentile 25 | .00 |  |  |
|  | Percentile 50 | 1.00 |  |  |
|  | Percentile 75 | 1.00 |  |  |
| Labeled Values | 0 | not correct answer | 60 | 45.8% |
|  | 1 | correct answer | 71 | 54.2% |

| **SS6.pre** | | |
| --- | --- | --- |
|  | | Value |
| Standard Attributes | Position | 27 |
|  | Label | SS6: Assessment of Pressure sores: 4 items |
|  | Type | Numeric |
|  | Format | F8.2 |
|  | Measurement | Scale |
|  | Role | Input |
| N | Valid | 131 |
|  | Missing | 0 |
| Central Tendency and Dispersion | Mean | .5592 |
|  | Standard Deviation | .24774 |
|  | Percentile 25 | .5000 |
|  | Percentile 50 | .5000 |
|  | Percentile 75 | .7500 |

| **k.18.pre** | | | | |
| --- | --- | --- | --- | --- |
|  | | Value | Count | Percent |
| Standard Attributes | Position | 28 |  |  |
|  | Label | Vitamins needed to maintain healthy skin, SS7: Nutrients and vitamins needed to prevent bed sore, 2 items |  |  |
|  | Type | Numeric |  |  |
|  | Format | F8 |  |  |
|  | Measurement | Scale |  |  |
|  | Role | Input |  |  |
| N | Valid | 131 |  |  |
|  | Missing | 0 |  |  |
| Central Tendency and Dispersion | Mean | .66 |  |  |
|  | Standard Deviation | .477 |  |  |
|  | Percentile 25 | .00 |  |  |
|  | Percentile 50 | 1.00 |  |  |
|  | Percentile 75 | 1.00 |  |  |
| Labeled Values | 0 | not correct answer | 45 | 34.4% |
|  | 1 | correct answer | 86 | 65.6% |

| **k.19.pre** | | | | |
| --- | --- | --- | --- | --- |
|  | | Value | Count | Percent |
| Standard Attributes | Position | 29 |  |  |
|  | Label | The nutrients needed to prevent bed sore in an elderly patients |  |  |
|  | Type | Numeric |  |  |
|  | Format | F8 |  |  |
|  | Measurement | Scale |  |  |
|  | Role | Input |  |  |
| N | Valid | 131 |  |  |
|  | Missing | 0 |  |  |
| Central Tendency and Dispersion | Mean | .70 |  |  |
|  | Standard Deviation | .459 |  |  |
|  | Percentile 25 | .00 |  |  |
|  | Percentile 50 | 1.00 |  |  |
|  | Percentile 75 | 1.00 |  |  |
| Labeled Values | 0 | not correct answer | 39 | 29.8% |
|  | 1 | correct answer | 92 | 70.2% |

| **SS7.pre** | | |
| --- | --- | --- |
|  | | Value |
| Standard Attributes | Position | 30 |
|  | Label | SS7: Nutrients and vitamins needed to prevent bed sore, 2 items |
|  | Type | Numeric |
|  | Format | F8.2 |
|  | Measurement | Scale |
|  | Role | Input |
| N | Valid | 131 |
|  | Missing | 0 |
| Central Tendency and Dispersion | Mean | .6794 |
|  | Standard Deviation | .31051 |
|  | Percentile 25 | .5000 |
|  | Percentile 50 | .5000 |
|  | Percentile 75 | 1.0000 |

| **k.20.pre** | | | | |
| --- | --- | --- | --- | --- |
|  | | Value | Count | Percent |
| Standard Attributes | Position | 31 |  |  |
|  | Label | Frequency of cleaning skin of a client with urinary or fecal incontinences, SS8: Nursing management of pressure sores: 14 items |  |  |
|  | Type | Numeric |  |  |
|  | Format | F8 |  |  |
|  | Measurement | Scale |  |  |
|  | Role | Input |  |  |
| N | Valid | 131 |  |  |
|  | Missing | 0 |  |  |
| Central Tendency and Dispersion | Mean | .69 |  |  |
|  | Standard Deviation | .462 |  |  |
|  | Percentile 25 | .00 |  |  |
|  | Percentile 50 | 1.00 |  |  |
|  | Percentile 75 | 1.00 |  |  |
| Labeled Values | 0 | not correct answer | 40 | 30.5% |
|  | 1 | correct answer | 91 | 69.5% |

| **k.21.pre** | | | | |
| --- | --- | --- | --- | --- |
|  | | Value | Count | Percent |
| Standard Attributes | Position | 32 |  |  |
|  | Label | The agent used for the skin cleaning |  |  |
|  | Type | Numeric |  |  |
|  | Format | F8 |  |  |
|  | Measurement | Scale |  |  |
|  | Role | Input |  |  |
| N | Valid | 131 |  |  |
|  | Missing | 0 |  |  |
| Central Tendency and Dispersion | Mean | .58 |  |  |
|  | Standard Deviation | .495 |  |  |
|  | Percentile 25 | .00 |  |  |
|  | Percentile 50 | 1.00 |  |  |
|  | Percentile 75 | 1.00 |  |  |
| Labeled Values | 0 | not correct answer | 55 | 42.0% |
|  | 1 | correct answer | 76 | 58.0% |

| **k.22.pre** | | | | |
| --- | --- | --- | --- | --- |
|  | | Value | Count | Percent |
| Standard Attributes | Position | 33 |  |  |
|  | Label | The frequency of changing position of a client confined to bed is once |  |  |
|  | Type | Numeric |  |  |
|  | Format | F8 |  |  |
|  | Measurement | Scale |  |  |
|  | Role | Input |  |  |
| N | Valid | 131 |  |  |
|  | Missing | 0 |  |  |
| Central Tendency and Dispersion | Mean | .55 |  |  |
|  | Standard Deviation | .499 |  |  |
|  | Percentile 25 | .00 |  |  |
|  | Percentile 50 | 1.00 |  |  |
|  | Percentile 75 | 1.00 |  |  |
| Labeled Values | 0 | not correct answer | 59 | 45.0% |
|  | 1 | correct answer | 72 | 55.0% |

| **k.23.pre** | | | | |
| --- | --- | --- | --- | --- |
|  | | Value | Count | Percent |
| Standard Attributes | Position | 34 |  |  |
|  | Label | The frequency of changing position of a client confined to chair is once |  |  |
|  | Type | Numeric |  |  |
|  | Format | F8 |  |  |
|  | Measurement | Scale |  |  |
|  | Role | Input |  |  |
| N | Valid | 131 |  |  |
|  | Missing | 0 |  |  |
| Central Tendency and Dispersion | Mean | .57 |  |  |
|  | Standard Deviation | .497 |  |  |
|  | Percentile 25 | .00 |  |  |
|  | Percentile 50 | 1.00 |  |  |
|  | Percentile 75 | 1.00 |  |  |
| Labeled Values | 0 | not correct answer | 56 | 42.7% |
|  | 1 | correct answer | 75 | 57.3% |

| **k.24.pre** | | | | |
| --- | --- | --- | --- | --- |
|  | | Value | Count | Percent |
| Standard Attributes | Position | 35 |  |  |
|  | Label | The main purposes of back care |  |  |
|  | Type | Numeric |  |  |
|  | Format | F8 |  |  |
|  | Measurement | Scale |  |  |
|  | Role | Input |  |  |
| N | Valid | 131 |  |  |
|  | Missing | 0 |  |  |
| Central Tendency and Dispersion | Mean | .63 |  |  |
|  | Standard Deviation | .486 |  |  |
|  | Percentile 25 | .00 |  |  |
|  | Percentile 50 | 1.00 |  |  |
|  | Percentile 75 | 1.00 |  |  |
| Labeled Values | 0 | not correct answer | 49 | 37.4% |
|  | 1 | correct answer | 82 | 62.6% |

| **k.25.pre** | | | | |
| --- | --- | --- | --- | --- |
|  | | Value | Count | Percent |
| Standard Attributes | Position | 36 |  |  |
|  | Label | The position used for back care |  |  |
|  | Type | Numeric |  |  |
|  | Format | F8 |  |  |
|  | Measurement | Scale |  |  |
|  | Role | Input |  |  |
| N | Valid | 131 |  |  |
|  | Missing | 0 |  |  |
| Central Tendency and Dispersion | Mean | .69 |  |  |
|  | Standard Deviation | .462 |  |  |
|  | Percentile 25 | .00 |  |  |
|  | Percentile 50 | 1.00 |  |  |
|  | Percentile 75 | 1.00 |  |  |
| Labeled Values | 0 | not correct answer | 40 | 30.5% |
|  | 1 | correct answer | 91 | 69.5% |

| **k.26.pre** | | | | |
| --- | --- | --- | --- | --- |
|  | | Value | Count | Percent |
| Standard Attributes | Position | 37 |  |  |
|  | Label | The agent used during massage to reduce friction is applying |  |  |
|  | Type | Numeric |  |  |
|  | Format | F8 |  |  |
|  | Measurement | Scale |  |  |
|  | Role | Input |  |  |
| N | Valid | 131 |  |  |
|  | Missing | 0 |  |  |
| Central Tendency and Dispersion | Mean | .40 |  |  |
|  | Standard Deviation | .491 |  |  |
|  | Percentile 25 | .00 |  |  |
|  | Percentile 50 | .00 |  |  |
|  | Percentile 75 | 1.00 |  |  |
| Labeled Values | 0 | not correct answer | 79 | 60.3% |
|  | 1 | correct answer | 52 | 39.7% |

| **k.27.pre** | | | | |
| --- | --- | --- | --- | --- |
|  | | Value | Count | Percent |
| Standard Attributes | Position | 38 |  |  |
|  | Label | Pressure relieving devices used to prevent pressure sore are |  |  |
|  | Type | Numeric |  |  |
|  | Format | F8 |  |  |
|  | Measurement | Scale |  |  |
|  | Role | Input |  |  |
| N | Valid | 131 |  |  |
|  | Missing | 0 |  |  |
| Central Tendency and Dispersion | Mean | .36 |  |  |
|  | Standard Deviation | .481 |  |  |
|  | Percentile 25 | .00 |  |  |
|  | Percentile 50 | .00 |  |  |
|  | Percentile 75 | 1.00 |  |  |
| Labeled Values | 0 | not correct answer | 84 | 64.1% |
|  | 1 | correct answer | 47 | 35.9% |

| **k.28.pre** | | | | |
| --- | --- | --- | --- | --- |
|  | | Value | Count | Percent |
| Standard Attributes | Position | 39 |  |  |
|  | Label | In supine position as a supportive device, pillows should be placed, |  |  |
|  | Type | Numeric |  |  |
|  | Format | F8 |  |  |
|  | Measurement | Scale |  |  |
|  | Role | Input |  |  |
| N | Valid | 131 |  |  |
|  | Missing | 0 |  |  |
| Central Tendency and Dispersion | Mean | .26 |  |  |
|  | Standard Deviation | .440 |  |  |
|  | Percentile 25 | .00 |  |  |
|  | Percentile 50 | .00 |  |  |
|  | Percentile 75 | 1.00 |  |  |
| Labeled Values | 0 | not correct answer | 97 | 74.0% |
|  | 1 | correct answer | 34 | 26.0% |

| **k.29.pre** | | | | |
| --- | --- | --- | --- | --- |
|  | | Value | Count | Percent |
| Standard Attributes | Position | 40 |  |  |
|  | Label | In lateral position, pillows should be placed, |  |  |
|  | Type | Numeric |  |  |
|  | Format | F8 |  |  |
|  | Measurement | Scale |  |  |
|  | Role | Input |  |  |
| N | Valid | 131 |  |  |
|  | Missing | 0 |  |  |
| Central Tendency and Dispersion | Mean | .29 |  |  |
|  | Standard Deviation | .456 |  |  |
|  | Percentile 25 | .00 |  |  |
|  | Percentile 50 | .00 |  |  |
|  | Percentile 75 | 1.00 |  |  |
| Labeled Values | 0 | not correct answer | 93 | 71.0% |
|  | 1 | correct answer | 38 | 29.0% |

| **k.30.pre** | | | | |
| --- | --- | --- | --- | --- |
|  | | Value | Count | Percent |
| Standard Attributes | Position | 41 |  |  |
|  | Label | An appropriate nursing care for managing mechanical load is, |  |  |
|  | Type | Numeric |  |  |
|  | Format | F8 |  |  |
|  | Measurement | Scale |  |  |
|  | Role | Input |  |  |
| N | Valid | 131 |  |  |
|  | Missing | 0 |  |  |
| Central Tendency and Dispersion | Mean | .63 |  |  |
|  | Standard Deviation | .484 |  |  |
|  | Percentile 25 | .00 |  |  |
|  | Percentile 50 | 1.00 |  |  |
|  | Percentile 75 | 1.00 |  |  |
| Labeled Values | 0 | not correct answer | 48 | 36.6% |
|  | 1 | correct answer | 83 | 63.4% |

| **k.31.pre** | | | | |
| --- | --- | --- | --- | --- |
|  | | Value | Count | Percent |
| Standard Attributes | Position | 42 |  |  |
|  | Label | An appropriate nursing activity to reduce friction |  |  |
|  | Type | Numeric |  |  |
|  | Format | F8 |  |  |
|  | Measurement | Scale |  |  |
|  | Role | Input |  |  |
| N | Valid | 131 |  |  |
|  | Missing | 0 |  |  |
| Central Tendency and Dispersion | Mean | .27 |  |  |
|  | Standard Deviation | .448 |  |  |
|  | Percentile 25 | .00 |  |  |
|  | Percentile 50 | .00 |  |  |
|  | Percentile 75 | 1.00 |  |  |
| Labeled Values | 0 | not correct answer | 95 | 72.5% |
|  | 1 | correct answer | 36 | 27.5% |

| **k.32.pre** | | | | |
| --- | --- | --- | --- | --- |
|  | | Value | Count | Percent |
| Standard Attributes | Position | 43 |  |  |
|  | Label | The nursing care for reducing shearing force |  |  |
|  | Type | Numeric |  |  |
|  | Format | F8 |  |  |
|  | Measurement | Scale |  |  |
|  | Role | Input |  |  |
| N | Valid | 131 |  |  |
|  | Missing | 0 |  |  |
| Central Tendency and Dispersion | Mean | .46 |  |  |
|  | Standard Deviation | .500 |  |  |
|  | Percentile 25 | .00 |  |  |
|  | Percentile 50 | .00 |  |  |
|  | Percentile 75 | 1.00 |  |  |
| Labeled Values | 0 | not correct answer | 71 | 54.2% |
|  | 1 | correct answer | 60 | 45.8% |

| **k.33.pre** | | | | |
| --- | --- | --- | --- | --- |
|  | | Value | Count | Percent |
| Standard Attributes | Position | 44 |  |  |
|  | Label | Exercise prevents pressure ulcer through, |  |  |
|  | Type | Numeric |  |  |
|  | Format | F8 |  |  |
|  | Measurement | Scale |  |  |
|  | Role | Input |  |  |
| N | Valid | 131 |  |  |
|  | Missing | 0 |  |  |
| Central Tendency and Dispersion | Mean | .39 |  |  |
|  | Standard Deviation | .489 |  |  |
|  | Percentile 25 | .00 |  |  |
|  | Percentile 50 | .00 |  |  |
|  | Percentile 75 | 1.00 |  |  |
| Labeled Values | 0 | not correct answer | 80 | 61.1% |
|  | 1 | correct answer | 51 | 38.9% |

| **SS8.pre** | | |
| --- | --- | --- |
|  | | Value |
| Standard Attributes | Position | 45 |
|  | Label | SS8: Nursing management of pressure sores: 14 items |
|  | Type | Numeric |
|  | Format | F8.2 |
|  | Measurement | Scale |
|  | Role | Input |
| N | Valid | 131 |
|  | Missing | 0 |
| Central Tendency and Dispersion | Mean | .4842 |
|  | Standard Deviation | .15732 |
|  | Percentile 25 | .3571 |
|  | Percentile 50 | .5000 |
|  | Percentile 75 | .5714 |

| **k.34.pre** | | | | |
| --- | --- | --- | --- | --- |
|  | | Value | Count | Percent |
| Standard Attributes | Position | 46 |  |  |
|  | Label | SS9: Complications of pressure sores, The most serious complication of pressure sore: 1 item |  |  |
|  | Type | Numeric |  |  |
|  | Format | F8 |  |  |
|  | Measurement | Scale |  |  |
|  | Role | Input |  |  |
| N | Valid | 131 |  |  |
|  | Missing | 0 |  |  |
| Central Tendency and Dispersion | Mean | .64 |  |  |
|  | Standard Deviation | .481 |  |  |
|  | Percentile 25 | .00 |  |  |
|  | Percentile 50 | 1.00 |  |  |
|  | Percentile 75 | 1.00 |  |  |
| Labeled Values | 0 | not correct answer | 47 | 35.9% |
|  | 1 | correct answer | 84 | 64.1% |

| **TotalPreKnowledge** | | |
| --- | --- | --- |
|  | | Value |
| Standard Attributes | Position | 47 |
|  | Label | Total Pre Knowledge |
|  | Type | Numeric |
|  | Format | F8.2 |
|  | Measurement | Scale |
|  | Role | Input |
| N | Valid | 131 |
|  | Missing | 0 |
| Central Tendency and Dispersion | Mean | 16.0992 |
|  | Standard Deviation | 4.59072 |
|  | Percentile 25 | 13.0000 |
|  | Percentile 50 | 15.0000 |
|  | Percentile 75 | 21.0000 |

| **VAR00001** | | |
| --- | --- | --- |
|  | | Value |
| Standard Attributes | Position | 48 |
|  | Label | ///////////////////////////////////////////////////////////////////////////////////////////////////////////////////////////////////////////////////// |
|  | Type | Numeric |
|  | Format | F8.2 |
|  | Measurement | Scale |
|  | Role | Input |
| N | Valid | 0 |
|  | Missing | 131 |
| Central Tendency and Dispersion | Mean | . |
|  | Standard Deviation | . |
|  | Percentile 25 | . |
|  | Percentile 50 | . |
|  | Percentile 75 | . |

| **a.pre1** | | | | |
| --- | --- | --- | --- | --- |
|  | | Value | Count | Percent |
| Standard Attributes | Position | 49 |  |  |
|  | Label | All patients are at potential risk of developing pressure ulcers. |  |  |
|  | Type | Numeric |  |  |
|  | Format | F8 |  |  |
|  | Measurement | Ordinal |  |  |
|  | Role | Input |  |  |
| Valid Values | 1 | strongly disagree | 67 | 51.1% |
|  | 2 | disagree | 0 | 0.0% |
|  | 3 | neutral | 12 | 9.2% |
|  | 4 | agree | 12 | 9.2% |
|  | 5 | strongly agree | 40 | 30.5% |

| **a.pre2** | | | | |
| --- | --- | --- | --- | --- |
|  | | Value | Count | Percent |
| Standard Attributes | Position | 50 |  |  |
|  | Label | Pressure ulcer prevention is time consuming for me to carry out. |  |  |
|  | Type | Numeric |  |  |
|  | Format | F8 |  |  |
|  | Measurement | Ordinal |  |  |
|  | Role | Input |  |  |
| Valid Values | 1 | strongly disagree | 41 | 31.3% |
|  | 2 | disagree | 32 | 24.4% |
|  | 3 | neutral | 24 | 18.3% |
|  | 4 | agree | 29 | 22.1% |
|  | 5 | strongly agree | 5 | 3.8% |

| **a.pre3** | | | | |
| --- | --- | --- | --- | --- |
|  | | Value | Count | Percent |
| Standard Attributes | Position | 51 |  |  |
|  | Label | In my opinion, patients tend not to get as many pressure ulcers nowadays. |  |  |
|  | Type | Numeric |  |  |
|  | Format | F8 |  |  |
|  | Measurement | Ordinal |  |  |
|  | Role | Input |  |  |
| Valid Values | 1 | strongly disagree | 76 | 58.0% |
|  | 2 | disagree | 0 | 0.0% |
|  | 3 | neutral | 4 | 3.1% |
|  | 4 | agree | 20 | 15.3% |
|  | 5 | strongly agree | 31 | 23.7% |

| **a.4pre** | | | | |
| --- | --- | --- | --- | --- |
|  | | Value | Count | Percent |
| Standard Attributes | Position | 52 |  |  |
|  | Label | I do not need to concern myself with pressure ulcer prevention in my practice. |  |  |
|  | Type | Numeric |  |  |
|  | Format | F8 |  |  |
|  | Measurement | Ordinal |  |  |
|  | Role | Input |  |  |
| Valid Values | 1 | strongly disagree | 85 | 64.9% |
|  | 2 | disagree | 1 | 0.8% |
|  | 3 | neutral | 8 | 6.1% |
|  | 4 | agree | 13 | 9.9% |
|  | 5 | strongly agree | 24 | 18.3% |

| **a.5pre** | | | | |
| --- | --- | --- | --- | --- |
|  | | Value | Count | Percent |
| Standard Attributes | Position | 53 |  |  |
|  | Label | Pressure ulcer treatment is a greater priority than pressure ulcer prevention |  |  |
|  | Type | Numeric |  |  |
|  | Format | F8 |  |  |
|  | Measurement | Ordinal |  |  |
|  | Role | Input |  |  |
| Valid Values | 1 | strongly disagree | 88 | 67.2% |
|  | 2 | disagree | 6 | 4.6% |
|  | 3 | neutral | 13 | 9.9% |
|  | 4 | agree | 21 | 16.0% |
|  | 5 | strongly agree | 3 | 2.3% |

| **a.6pre** | | | | |
| --- | --- | --- | --- | --- |
|  | | Value | Count | Percent |
| Standard Attributes | Position | 54 |  |  |
|  | Label | Continuous assessment of patients will give an accurate account of their pressure ulcer risk |  |  |
|  | Type | Numeric |  |  |
|  | Format | F8 |  |  |
|  | Measurement | Ordinal |  |  |
|  | Role | Input |  |  |
| Valid Values | 1 | strongly disagree | 92 | 70.2% |
|  | 2 | disagree | 0 | 0.0% |
|  | 3 | neutral | 0 | 0.0% |
|  | 4 | agree | 15 | 11.5% |
|  | 5 | strongly agree | 24 | 18.3% |

| **a.7pre** | | | | |
| --- | --- | --- | --- | --- |
|  | | Value | Count | Percent |
| Standard Attributes | Position | 55 |  |  |
|  | Label | Most pressure ulcers can be avoided. |  |  |
|  | Type | Numeric |  |  |
|  | Format | F8 |  |  |
|  | Measurement | Ordinal |  |  |
|  | Role | Input |  |  |
| Valid Values | 1 | strongly disagree | 69 | 52.7% |
|  | 2 | disagree | 5 | 3.8% |
|  | 3 | neutral | 12 | 9.2% |
|  | 4 | agree | 15 | 11.5% |
|  | 5 | strongly agree | 30 | 22.9% |

| **a.8pre** | | | | |
| --- | --- | --- | --- | --- |
|  | | Value | Count | Percent |
| Standard Attributes | Position | 56 |  |  |
|  | Label | I am less interested in pressure ulcer prevention than other aspects of care. |  |  |
|  | Type | Numeric |  |  |
|  | Format | F8 |  |  |
|  | Measurement | Ordinal |  |  |
|  | Role | Input |  |  |
| Valid Values | 1 | strongly disagree | 76 | 58.0% |
|  | 2 | disagree | 1 | 0.8% |
|  | 3 | neutral | 10 | 7.6% |
|  | 4 | agree | 25 | 19.1% |
|  | 5 | strongly agree | 19 | 14.5% |

| **a.9pre** | | | | |
| --- | --- | --- | --- | --- |
|  | | Value | Count | Percent |
| Standard Attributes | Position | 57 |  |  |
|  | Label | My clinical judgment is better than any pressure ulcer risk assessment tool available to me. |  |  |
|  | Type | Numeric |  |  |
|  | Format | F8 |  |  |
|  | Measurement | Ordinal |  |  |
|  | Role | Input |  |  |
| Valid Values | 1 | strongly disagree | 72 | 55.0% |
|  | 2 | disagree | 14 | 10.7% |
|  | 3 | neutral | 23 | 17.6% |
|  | 4 | agree | 11 | 8.4% |
|  | 5 | strongly agree | 11 | 8.4% |

| **a.10pre** | | | | |
| --- | --- | --- | --- | --- |
|  | | Value | Count | Percent |
| Standard Attributes | Position | 58 |  |  |
|  | Label | In comparison with other areas of care, pressure ulcer prevention is a low priority for me. |  |  |
|  | Type | Numeric |  |  |
|  | Format | F8 |  |  |
|  | Measurement | Ordinal |  |  |
|  | Role | Input |  |  |
| Valid Values | 1 | strongly disagree | 69 | 52.7% |
|  | 2 | disagree | 1 | 0.8% |
|  | 3 | neutral | 15 | 11.5% |
|  | 4 | agree | 7 | 5.3% |
|  | 5 | strongly agree | 39 | 29.8% |

| **a.11pre** | | | | |
| --- | --- | --- | --- | --- |
|  | | Value | Count | Percent |
| Standard Attributes | Position | 59 |  |  |
|  | Label | Pressure ulcer risk assessment should be regularly carried out on all patients during their stay in the hospital. |  |  |
|  | Type | Numeric |  |  |
|  | Format | F8 |  |  |
|  | Measurement | Ordinal |  |  |
|  | Role | Input |  |  |
| Valid Values | 1 | strongly disagree | 54 | 41.2% |
|  | 2 | disagree | 14 | 10.7% |
|  | 3 | neutral | 16 | 12.2% |
|  | 4 | agree | 23 | 17.6% |
|  | 5 | strongly agree | 24 | 18.3% |

| **TotalAttitPre** | | |
| --- | --- | --- |
|  | | Value |
| Standard Attributes | Position | 60 |
|  | Label | Average of Total Attitude Pretest |
|  | Type | Numeric |
|  | Format | F8.2 |
|  | Measurement | Scale |
|  | Role | Input |
| N | Valid | 131 |
|  | Missing | 0 |
| Central Tendency and Dispersion | Mean | 2.3331 |
|  | Standard Deviation | .49992 |
|  | Percentile 25 | 2.0000 |
|  | Percentile 50 | 2.3636 |
|  | Percentile 75 | 2.6364 |

| **VAR00002** | | |
| --- | --- | --- |
|  | | Value |
| Standard Attributes | Position | 61 |
|  | Label | ///////////////////////////////////////////////////////////////////////////////////////////////////////////////////////////////////////////////////////////// |
|  | Type | Numeric |
|  | Format | F8.2 |
|  | Measurement | Scale |
|  | Role | Input |
| N | Valid | 0 |
|  | Missing | 131 |
| Central Tendency and Dispersion | Mean | . |
|  | Standard Deviation | . |
|  | Percentile 25 | . |
|  | Percentile 50 | . |
|  | Percentile 75 | . |

| **p.ractice.1.pre** | | | | |
| --- | --- | --- | --- | --- |
|  | | Value | Count | Percent |
| Standard Attributes | Position | 62 |  |  |
|  | Label | I Observe how other nurses assess the risk factors |  |  |
|  | Type | Numeric |  |  |
|  | Format | F8 |  |  |
|  | Measurement | Nominal |  |  |
|  | Role | Input |  |  |
| Valid Values | 0 | not done | 63 | 48.1% |
|  | 1 | done | 68 | 51.9% |

| **p.ractice..2.pre** | | | | |
| --- | --- | --- | --- | --- |
|  | | Value | Count | Percent |
| Standard Attributes | Position | 63 |  |  |
|  | Label | I identify common contributing factors |  |  |
|  | Type | Numeric |  |  |
|  | Format | F8 |  |  |
|  | Measurement | Nominal |  |  |
|  | Role | Input |  |  |
| Valid Values | 0 | not done | 48 | 36.6% |
|  | 1 | done | 83 | 63.4% |

| **p.ractice..3.pre** | | | | |
| --- | --- | --- | --- | --- |
|  | | Value | Count | Percent |
| Standard Attributes | Position | 64 |  |  |
|  | Label | I do a skin assessment |  |  |
|  | Type | Numeric |  |  |
|  | Format | F8 |  |  |
|  | Measurement | Nominal |  |  |
|  | Role | Input |  |  |
| Valid Values | 0 | not done | 53 | 40.5% |
|  | 1 | done | 78 | 59.5% |

| **p.ractice.4.pre** | | | | |
| --- | --- | --- | --- | --- |
|  | | Value | Count | Percent |
| Standard Attributes | Position | 65 |  |  |
|  | Label | I use risk assessment scale |  |  |
|  | Type | Numeric |  |  |
|  | Format | F8 |  |  |
|  | Measurement | Nominal |  |  |
|  | Role | Input |  |  |
| Valid Values | 0 | not done | 104 | 79.4% |
|  | 1 | done | 27 | 20.6% |

| **p.ractice..5.pre** | | | | |
| --- | --- | --- | --- | --- |
|  | | Value | Count | Percent |
| Standard Attributes | Position | 66 |  |  |
|  | Label | I document all data |  |  |
|  | Type | Numeric |  |  |
|  | Format | F8 |  |  |
|  | Measurement | Nominal |  |  |
|  | Role | Input |  |  |
| Valid Values | 0 | not done | 23 | 17.6% |
|  | 1 | done | 108 | 82.4% |

| **p.ractice..6.pre** | | | | |
| --- | --- | --- | --- | --- |
|  | | Value | Count | Percent |
| Standard Attributes | Position | 67 |  |  |
|  | Label | I assess and provide management of pain |  |  |
|  | Type | Numeric |  |  |
|  | Format | F8 |  |  |
|  | Measurement | Nominal |  |  |
|  | Role | Input |  |  |
| Valid Values | 0 | not done | 124 | 94.7% |
|  | 1 | done | 7 | 5.3% |

| **p.ractice.7.pre** | | | | |
| --- | --- | --- | --- | --- |
|  | | Value | Count | Percent |
| Standard Attributes | Position | 68 |  |  |
|  | Label | I perform skin care as a routine work |  |  |
|  | Type | Numeric |  |  |
|  | Format | F8 |  |  |
|  | Measurement | Nominal |  |  |
|  | Role | Input |  |  |
| Valid Values | 0 | not done | 55 | 42.0% |
|  | 1 | done | 76 | 58.0% |

| **p.ractice..8.pre** | | | | |
| --- | --- | --- | --- | --- |
|  | | Value | Count | Percent |
| Standard Attributes | Position | 69 |  |  |
|  | Label | I place the pillow under the patient’s leg |  |  |
|  | Type | Numeric |  |  |
|  | Format | F8 |  |  |
|  | Measurement | Nominal |  |  |
|  | Role | Input |  |  |
| Valid Values | 0 | not done | 89 | 67.9% |
|  | 1 | done | 42 | 32.1% |

| **p.ractice..9.pre** | | | | |
| --- | --- | --- | --- | --- |
|  | | Value | Count | Percent |
| Standard Attributes | Position | 70 |  |  |
|  | Label | I use water filled glove under the patient’s leg |  |  |
|  | Type | Numeric |  |  |
|  | Format | F8 |  |  |
|  | Measurement | Nominal |  |  |
|  | Role | Input |  |  |
| Valid Values | 0 | not done | 25 | 19.1% |
|  | 1 | done | 106 | 80.9% |

| **p.ractice..10.pre** | | | | |
| --- | --- | --- | --- | --- |
|  | | Value | Count | Percent |
| Standard Attributes | Position | 71 |  |  |
|  | Label | I use or advice caregiver to use creams or oils |  |  |
|  | Type | Numeric |  |  |
|  | Format | F8 |  |  |
|  | Measurement | Nominal |  |  |
|  | Role | Input |  |  |
| Valid Values | 0 | not done | 51 | 38.9% |
|  | 1 | done | 80 | 61.1% |

| **p.ractice.11.pre** | | | | |
| --- | --- | --- | --- | --- |
|  | | Value | Count | Percent |
| Standard Attributes | Position | 72 |  |  |
|  | Label | I pay more attention to pressure points |  |  |
|  | Type | Numeric |  |  |
|  | Format | F8 |  |  |
|  | Measurement | Nominal |  |  |
|  | Role | Input |  |  |
| Valid Values | 0 | not done | 75 | 57.3% |
|  | 1 | done | 56 | 42.7% |

| **p.ractice..12.pre** | | | | |
| --- | --- | --- | --- | --- |
|  | | Value | Count | Percent |
| Standard Attributes | Position | 73 |  |  |
|  | Label | I perform lab tests |  |  |
|  | Type | Numeric |  |  |
|  | Format | F8 |  |  |
|  | Measurement | Nominal |  |  |
|  | Role | Input |  |  |
| Valid Values | 0 | not done | 127 | 96.9% |
|  | 1 | done | 4 | 3.1% |

| **p.ractice..13.pre** | | | | |
| --- | --- | --- | --- | --- |
|  | | Value | Count | Percent |
| Standard Attributes | Position | 74 |  |  |
|  | Label | I provide vitamin and food |  |  |
|  | Type | Numeric |  |  |
|  | Format | F8 |  |  |
|  | Measurement | Nominal |  |  |
|  | Role | Input |  |  |
| Valid Values | 0 | not done | 22 | 16.8% |
|  | 1 | done | 109 | 83.2% |

| **p.ractice.14.pre** | | | | |
| --- | --- | --- | --- | --- |
|  | | Value | Count | Percent |
| Standard Attributes | Position | 75 |  |  |
|  | Label | I monitor a protein and calorie diet |  |  |
|  | Type | Numeric |  |  |
|  | Format | F8 |  |  |
|  | Measurement | Nominal |  |  |
|  | Role | Input |  |  |
| Valid Values | 0 | not done | 108 | 82.4% |
|  | 1 | done | 23 | 17.6% |

| **p.ractice.15.pre** | | | | |
| --- | --- | --- | --- | --- |
|  | | Value | Count | Percent |
| Standard Attributes | Position | 76 |  |  |
|  | Label | I avoid dragging |  |  |
|  | Type | Numeric |  |  |
|  | Format | F8 |  |  |
|  | Measurement | Nominal |  |  |
|  | Role | Input |  |  |
| Valid Values | 0 | not done | 84 | 64.1% |
|  | 1 | done | 47 | 35.9% |

| **p.ractice..16.pre** | | | | |
| --- | --- | --- | --- | --- |
|  | | Value | Count | Percent |
| Standard Attributes | Position | 77 |  |  |
|  | Label | I always use a special mattress |  |  |
|  | Type | Numeric |  |  |
|  | Format | F8 |  |  |
|  | Measurement | Nominal |  |  |
|  | Role | Input |  |  |
| Valid Values | 0 | not done | 80 | 61.1% |
|  | 1 | done | 51 | 38.9% |

| **p.ractice..17.pre** | | | | |
| --- | --- | --- | --- | --- |
|  | | Value | Count | Percent |
| Standard Attributes | Position | 78 |  |  |
|  | Label | I avoid massage |  |  |
|  | Type | Numeric |  |  |
|  | Format | F8 |  |  |
|  | Measurement | Nominal |  |  |
|  | Role | Input |  |  |
| Valid Values | 0 | not done | 109 | 83.2% |
|  | 1 | done | 22 | 16.8% |

| **p.ractice..18.pre** | | | | |
| --- | --- | --- | --- | --- |
|  | | Value | Count | Percent |
| Standard Attributes | Position | 79 |  |  |
|  | Label | I avoid using donut – shape (ring) cushion |  |  |
|  | Type | Numeric |  |  |
|  | Format | F8 |  |  |
|  | Measurement | Nominal |  |  |
|  | Role | Input |  |  |
| Valid Values | 0 | not done | 67 | 51.1% |
|  | 1 | done | 64 | 48.9% |

| **p.ractice..19.pre** | | | | |
| --- | --- | --- | --- | --- |
|  | | Value | Count | Percent |
| Standard Attributes | Position | 80 |  |  |
|  | Label | I turn a patient position every two hours. |  |  |
|  | Type | Numeric |  |  |
|  | Format | F8 |  |  |
|  | Measurement | Nominal |  |  |
|  | Role | Input |  |  |
| Valid Values | 0 | not done | 66 | 50.4% |
|  | 1 | done | 65 | 49.6% |

| **p.ractice.20.pre** | | | | |
| --- | --- | --- | --- | --- |
|  | | Value | Count | Percent |
| Standard Attributes | Position | 81 |  |  |
|  | Label | I put pillows under the patient’s leg ankle |  |  |
|  | Type | Numeric |  |  |
|  | Format | F8 |  |  |
|  | Measurement | Nominal |  |  |
|  | Role | Input |  |  |
| Valid Values | 0 | not done | 63 | 48.1% |
|  | 1 | done | 68 | 51.9% |

| **TotalPracPre** | | |
| --- | --- | --- |
|  | | Value |
| Standard Attributes | Position | 82 |
|  | Label | Total Practice Pretest |
|  | Type | Numeric |
|  | Format | F8.2 |
|  | Measurement | Scale |
|  | Role | Input |
| N | Valid | 131 |
|  | Missing | 0 |
| Central Tendency and Dispersion | Mean | 9.0382 |
|  | Standard Deviation | 3.37959 |
|  | Percentile 25 | 6.0000 |
|  | Percentile 50 | 9.0000 |
|  | Percentile 75 | 12.0000 |

| **VAR00021** | | |
| --- | --- | --- |
|  | | Value |
| Standard Attributes | Position | 83 |
|  | Label | //////////////////////////////////////////////////////////////////////////////////////////////////////////////////////////////////////////////////////////// |
|  | Type | Numeric |
|  | Format | F8.2 |
|  | Measurement | Scale |
|  | Role | Input |
| N | Valid | 0 |
|  | Missing | 131 |
| Central Tendency and Dispersion | Mean | . |
|  | Standard Deviation | . |
|  | Percentile 25 | . |
|  | Percentile 50 | . |
|  | Percentile 75 | . |

| **pre.EX.1** | | | | |
| --- | --- | --- | --- | --- |
|  | | Value | Count | Percent |
| Standard Attributes | Position | 84 |  |  |
|  | Label | I feel mentally exhausted |  |  |
|  | Type | Numeric |  |  |
|  | Format | F8.2 |  |  |
|  | Measurement | Ordinal |  |  |
|  | Role | Input |  |  |
| Valid Values | 1.00 | never | 4 | 3.1% |
|  | 2.00 | rarely | 45 | 34.4% |
|  | 3.00 | sometimes | 23 | 17.6% |
|  | 4.00 | often | 29 | 22.1% |
|  | 5.00 | always | 30 | 22.9% |

| **pre.EX.2** | | | | |
| --- | --- | --- | --- | --- |
|  | | Value | Count | Percent |
| Standard Attributes | Position | 85 |  |  |
|  | Label | At the end of the day, I find it hard to recover my energy |  |  |
|  | Type | Numeric |  |  |
|  | Format | F8.2 |  |  |
|  | Measurement | Ordinal |  |  |
|  | Role | Input |  |  |
| Valid Values | 1.00 | never | 17 | 13.0% |
|  | 2.00 | rarely | 2 | 1.5% |
|  | 3.00 | sometimes | 57 | 43.5% |
|  | 4.00 | often | 34 | 26.0% |
|  | 5.00 | always | 21 | 16.0% |

| **pre.EX.3** | | | | |
| --- | --- | --- | --- | --- |
|  | | Value | Count | Percent |
| Standard Attributes | Position | 86 |  |  |
|  | Label | I feel physically exhausted |  |  |
|  | Type | Numeric |  |  |
|  | Format | F8.2 |  |  |
|  | Measurement | Ordinal |  |  |
|  | Role | Input |  |  |
| Valid Values | 1.00 | never | 16 | 12.2% |
|  | 2.00 | rarely | 6 | 4.6% |
|  | 3.00 | sometimes | 36 | 27.5% |
|  | 4.00 | often | 49 | 37.4% |
|  | 5.00 | always | 24 | 18.3% |

| **Pre.Ex** | | |
| --- | --- | --- |
|  | | Value |
| Standard Attributes | Position | 87 |
|  | Label | Exhaustion subscale |
|  | Type | Numeric |
|  | Format | F8.2 |
|  | Measurement | Scale |
|  | Role | Input |
| N | Valid | 131 |
|  | Missing | 0 |
| Central Tendency and Dispersion | Mean | 3.3435 |
|  | Standard Deviation | .77010 |
|  | Percentile 25 | 3.0000 |
|  | Percentile 50 | 3.3333 |
|  | Percentile 75 | 3.6667 |

| **pre.MD.1** | | | | |
| --- | --- | --- | --- | --- |
|  | | Value | Count | Percent |
| Standard Attributes | Position | 88 |  |  |
|  | Label | I struggle to find any enthusiasm for my work |  |  |
|  | Type | Numeric |  |  |
|  | Format | F8.2 |  |  |
|  | Measurement | Ordinal |  |  |
|  | Role | Input |  |  |
| Valid Values | 1.00 | never | 5 | 3.8% |
|  | 2.00 | rarely | 26 | 19.8% |
|  | 3.00 | sometimes | 52 | 39.7% |
|  | 4.00 | often | 27 | 20.6% |
|  | 5.00 | always | 21 | 16.0% |

| **pre.MD.2** | | | | |
| --- | --- | --- | --- | --- |
|  | | Value | Count | Percent |
| Standard Attributes | Position | 89 |  |  |
|  | Label | I feel a strong aversion towards my job |  |  |
|  | Type | Numeric |  |  |
|  | Format | F8.2 |  |  |
|  | Measurement | Ordinal |  |  |
|  | Role | Input |  |  |
| Valid Values | 1.00 | never | 16 | 12.2% |
|  | 2.00 | rarely | 16 | 12.2% |
|  | 3.00 | sometimes | 43 | 32.8% |
|  | 4.00 | often | 34 | 26.0% |
|  | 5.00 | always | 22 | 16.8% |

| **pre.MD.3** | | | | |
| --- | --- | --- | --- | --- |
|  | | Value | Count | Percent |
| Standard Attributes | Position | 90 |  |  |
|  | Label | I am cynical about what my work means to others |  |  |
|  | Type | Numeric |  |  |
|  | Format | F8.2 |  |  |
|  | Measurement | Ordinal |  |  |
|  | Role | Input |  |  |
| Valid Values | 1.00 | never | 9 | 6.9% |
|  | 2.00 | rarely | 5 | 3.8% |
|  | 3.00 | sometimes | 35 | 26.7% |
|  | 4.00 | often | 54 | 41.2% |
|  | 5.00 | always | 28 | 21.4% |

| **Pre.MD** | | |
| --- | --- | --- |
|  | | Value |
| Standard Attributes | Position | 91 |
|  | Label | Mental distance subscale |
|  | Type | Numeric |
|  | Format | F8.2 |
|  | Measurement | Scale |
|  | Role | Input |
| N | Valid | 131 |
|  | Missing | 0 |
| Central Tendency and Dispersion | Mean | 3.3817 |
|  | Standard Deviation | .75234 |
|  | Percentile 25 | 3.0000 |
|  | Percentile 50 | 3.0000 |
|  | Percentile 75 | 3.6667 |

| **pre.CI.1** | | | | |
| --- | --- | --- | --- | --- |
|  | | Value | Count | Percent |
| Standard Attributes | Position | 92 |  |  |
|  | Label | I have trouble staying focused |  |  |
|  | Type | Numeric |  |  |
|  | Format | F8.2 |  |  |
|  | Measurement | Ordinal |  |  |
|  | Role | Input |  |  |
| Valid Values | 1.00 | never | 8 | 6.1% |
|  | 2.00 | rarely | 1 | 0.8% |
|  | 3.00 | sometimes | 45 | 34.4% |
|  | 4.00 | often | 52 | 39.7% |
|  | 5.00 | always | 25 | 19.1% |

| **pre.CI.2** | | | | |
| --- | --- | --- | --- | --- |
|  | | Value | Count | Percent |
| Standard Attributes | Position | 93 |  |  |
|  | Label | I have trouble concentrating |  |  |
|  | Type | Numeric |  |  |
|  | Format | F8.2 |  |  |
|  | Measurement | Ordinal |  |  |
|  | Role | Input |  |  |
| Valid Values | 1.00 | never | 4 | 3.1% |
|  | 2.00 | rarely | 12 | 9.2% |
|  | 3.00 | sometimes | 33 | 25.2% |
|  | 4.00 | often | 57 | 43.5% |
|  | 5.00 | always | 25 | 19.1% |

| **pre.CI.3** | | | | |
| --- | --- | --- | --- | --- |
|  | | Value | Count | Percent |
| Standard Attributes | Position | 94 |  |  |
|  | Label | I make mistakes because I have my mind on other things |  |  |
|  | Type | Numeric |  |  |
|  | Format | F8.2 |  |  |
|  | Measurement | Ordinal |  |  |
|  | Role | Input |  |  |
| Valid Values | 1.00 | never | 8 | 6.1% |
|  | 2.00 | rarely | 14 | 10.7% |
|  | 3.00 | sometimes | 46 | 35.1% |
|  | 4.00 | often | 26 | 19.8% |
|  | 5.00 | always | 37 | 28.2% |

| **Pre.CI** | | |
| --- | --- | --- |
|  | | Value |
| Standard Attributes | Position | 95 |
|  | Label | Cognitive impairment subscale |
|  | Type | Numeric |
|  | Format | F8.2 |
|  | Measurement | Scale |
|  | Role | Input |
| N | Valid | 131 |
|  | Missing | 0 |
| Central Tendency and Dispersion | Mean | 3.6158 |
|  | Standard Deviation | .77842 |
|  | Percentile 25 | 3.0000 |
|  | Percentile 50 | 3.6667 |
|  | Percentile 75 | 4.0000 |

| **pre.EI.1** | | | | |
| --- | --- | --- | --- | --- |
|  | | Value | Count | Percent |
| Standard Attributes | Position | 96 |  |  |
|  | Label | I feel unable to control my emotions |  |  |
|  | Type | Numeric |  |  |
|  | Format | F8.2 |  |  |
|  | Measurement | Ordinal |  |  |
|  | Role | Input |  |  |
| Valid Values | 1.00 | never | 4 | 3.1% |
|  | 2.00 | rarely | 6 | 4.6% |
|  | 3.00 | sometimes | 41 | 31.3% |
|  | 4.00 | often | 54 | 41.2% |
|  | 5.00 | always | 26 | 19.8% |

| **pre.EI.2** | | | | |
| --- | --- | --- | --- | --- |
|  | | Value | Count | Percent |
| Standard Attributes | Position | 97 |  |  |
|  | Label | I do not recognize myself in the way I react emotionally |  |  |
|  | Type | Numeric |  |  |
|  | Format | F8.2 |  |  |
|  | Measurement | Ordinal |  |  |
|  | Role | Input |  |  |
| Valid Values | 1.00 | never | 8 | 6.1% |
|  | 2.00 | rarely | 11 | 8.4% |
|  | 3.00 | sometimes | 48 | 36.6% |
|  | 4.00 | often | 41 | 31.3% |
|  | 5.00 | always | 23 | 17.6% |

| **pre.EI.3** | | | | |
| --- | --- | --- | --- | --- |
|  | | Value | Count | Percent |
| Standard Attributes | Position | 98 |  |  |
|  | Label | I may overreact unintentionally |  |  |
|  | Type | Numeric |  |  |
|  | Format | F8.2 |  |  |
|  | Measurement | Ordinal |  |  |
|  | Role | Input |  |  |
| Valid Values | 1.00 | never | 4 | 3.1% |
|  | 2.00 | rarely | 6 | 4.6% |
|  | 3.00 | sometimes | 48 | 36.6% |
|  | 4.00 | often | 48 | 36.6% |
|  | 5.00 | always | 25 | 19.1% |

| **Pre.EI** | | |
| --- | --- | --- |
|  | | Value |
| Standard Attributes | Position | 99 |
|  | Label | Emotional impairment subscae |
|  | Type | Numeric |
|  | Format | F8.2 |
|  | Measurement | Scale |
|  | Role | Input |
| N | Valid | 131 |
|  | Missing | 0 |
| Central Tendency and Dispersion | Mean | 3.6005 |
|  | Standard Deviation | .90904 |
|  | Percentile 25 | 3.0000 |
|  | Percentile 50 | 3.6667 |
|  | Percentile 75 | 4.0000 |

| **TotalBurnoutPre** | | |
| --- | --- | --- |
|  | | Value |
| Standard Attributes | Position | 100 |
|  | Label | Mean Burnout Scale Pretest |
|  | Type | Numeric |
|  | Format | F8.2 |
|  | Measurement | Scale |
|  | Role | Input |
| N | Valid | 131 |
|  | Missing | 0 |
| Central Tendency and Dispersion | Mean | 3.4854 |
|  | Standard Deviation | .65212 |
|  | Percentile 25 | 3.0833 |
|  | Percentile 50 | 3.4167 |
|  | Percentile 75 | 3.7500 |

| **VAR00036** | | |
| --- | --- | --- |
|  | | Value |
| Standard Attributes | Position | 101 |
|  | Label | ****************************************************************************************************************************** |
|  | Type | Numeric |
|  | Format | F9.2 |
|  | Measurement | Scale |
|  | Role | Input |
| N | Valid | 0 |
|  | Missing | 131 |
| Central Tendency and Dispersion | Mean | . |
|  | Standard Deviation | . |
|  | Percentile 25 | . |
|  | Percentile 50 | . |
|  | Percentile 75 | . |
