# Supplementary material for: Nurses’ knowledge, attitudes, and practices in pressure ulcer prevention in intensive care units: associations with burnout
Source: PeerJ. 2026 May 14;14:e21250. doi: 10.7717/peerj.21250 (PMC13180348; doi:10.7717/peerj.21250)
Supplement: Supplemental Information 5 [file peerj-14-21250-s005.docx]

**Questionnaire regarding Nurses' Knowledge, Attitudes, and Practices in Pressure Ulcer Prevention in Intensive Care Units: Associations with Burnout**

**Age**

less than 30 years

30 to less than 40 years

40 to less than 50 years

50 to 60 years

**Gender**:

Men

Women

**Marital Status:**

Married

Single

Widower

Divorced

**Education:**

Diploma nurse

Technical diploma in nursing

Bachelor of Nursing

**Experience years:**

Less than 5 years

5 to 10 years

More than 10 years

**Knowledge Regarding Preventing Pressure Ulcers.**

Instruction: Please tick mark ( ) on your answer in the bracket as indicated.

**1. The definition of a pressure ulcer is:**

a. Localized injury caused by unrelieved pressure. ( )

b. Damage to the skin. ( )

c. Inadequate blood supply. ( )

d. Necrosis. ( )

2**. Other nomenclature for pressure ulcer:**

a. Skin tear. ( )

b. Break in the tissue. ( )

c. Decubitus ulcer. ( )

d. Lesion. ( )

**3. The main cause of pressure ulcers is:**

a. Advanced age. ( )

b. impaired blood circulation. ( )

c. pressure. ( )

d. Unconsciousness. ( )

**4. The commonest age for developing pressure ulcers is:**

a. Adulthood. ( )

b. Middle age. ( )

c. Old age. ( )

d. Elderly. ( )

**5. Clients with urinary or fecal incontinence develop pressure ulcers due to:**

a. Unrelieved pressure. ( )

b. Moisture. ( )

c. Friction. ( )

d. Bacterial infection. ( )

6**. Client under shearing force developed a pressure ulcer due to:**

a. Friction and gravity. ( )

b. Pressure and gravity. ( )

c. Moisture and pressure. ( )

d. Friction and pressure. ( )

**7. Development of pressure ulcers from the wrong method of using a bedpan occurs due to:**

a. Moisture. ( )

b. Gravity. ( )

c. Shearing force. ( )

d. Friction ( )

**8. The point of highest pressure when the client is in the Lateral position:**

a. Greater trochanter and hip. ( )

b. Ears and back of scapula. ( )

c. Abdomen and thigh. ( )

d. Elbows and heels. ( )

9. **The point of highest pressure in the supine position is:**

a. Nape of neck. ( )

b. Elbows. ( )

c. Thigh. ( )

d. Sacral region. ( )

1**0. The point of highest pressure in a sitting position is:**

a. Shoulders. ( )

b. Back. ( )

c. Buttocks. ( )

d. Sole. ( )

**11. The first sign of pressure ulcer development is:**

a. Open ulcer. ( )

b. Burning sensation. ( )

c. Blister and bluish discoloration in the skin. ( )

d. Redness. ( )

**12. The following are symptoms of stage III pressure ulcer**:

a. Open ulcer with a red-pink wound bed. ( )

b. Subcutaneous fat may be visible. ( )

c. Bone and tendon are exposed. ( )

d. Shiny or dry shallow ulcer without slough or bruising. ( )

**13. The symptom of the unstageable/ Unclassified category of pressure ulcer is:**

a. Open ulcer. ( )

b. Partial thickening. ( )

c. Actual depth of the ulcer is obscured by eschar depth cannot be

determined. ( )

d. Deep tissue injury. ( )

**14. The appropriate ways for the assessment of high-risk pressure ulcers**

**development is:**

a. Risk assessment scale. ( )

b. Clinical judgment. ( )

c. Physician’s order. ( )

d. Laboratory test. ( )

**15. The appropriate scale for pressure ulcer risk assessment is:**

a. Head-to-toe scale. ( )

b. Braden scale. ( )

c. Glasgow coma scale. ( )

d. Role of nine-point scale. ( )

**16. Frequency of skin assessment is:**

a. Daily. ( )

b. Every two days. ( )

c. Every three days. ( )

d. Weekly. ( )

**17. Area for having more attention, while performing skin assessment is:**

a. Bony prominence. ( )

b. Skin folds. ( )

c. The back. ( )

d. Skin over flesh. ( )

**18. Vitamins needed to maintain healthy skin are:**

a. Vitamin D. ( )

b. Vitamin C &E. ( )

c. Vitamin K. ( )

d. Vitamin B. ( )

**19. The nutrients needed to prevent bed ulcers in elderly patients:**

a. High fats. ( )

b. High protein and high calorie. ( )

c. High fiber diet. ( )

d. High fats and high fiber. ( )

**20. Frequency of cleaning the skin of a client with urinary or fecal incontinence:**

a. Every half an hour. ( )

b. Once in two hours at a regular interval. ( )

c. Once in four hours at a regular interval. ( )

d. During the time of soiling and at regular intervals. ( )

**21. The agent used for skin cleaning is**

a. Herbal soap. ( )

b. Cream-based soap. ( )

c. Antiseptic lotion. ( )

d. Mild detergent. ( )

**22. The frequency of changing the position of a client confined to bed is once in:**

a. Every two hours. ( )

b. Every three hours. ( )

c. Every four hours. ( )

d. Every six hours. ( )

**23. The frequency of changing the position of a client confined to a chair is once in:**

a. hourly. ( )

b. Every two hours. ( )

c. Every three hours. ( )

d. Every four hours. ( )

**24. The main purposes of back care are:**

a. Improve circulation. ( )

b. Prevent skin breakdown. ( )

c. Promote soothing effect. ( )

d. All above. ( )

**25. The position used for back care is:**

a. modified lateral position. ( )

b. flowers position. ( )

c. Prone position. ( )

d. sitting or lateral position. ( )

**26. The agent used during massage to reduce friction is applying:**

a. Lubricant or lotion. ( )

b. Ointment. ( )

c. Talk powder. ( )

d. Iodine. ( )

**27. Pressure-relieving devices used to prevent pressure ulcers are:**

a. Air mattress. ( )

b. Cushions. ( )

c. Water mattress. ( )

d. All above. ( )

**28. In the supine position, as a supportive device, pillows should be placed:**

a. Under head, hands, arms, lower back, and lower legs. ( )

b. Under the head, between the legs, and back. ( )

c. Under the head and shoulder and between the legs. ( )

d. Under the hands, shoulder, back, and legs. ( )

**29. In the lateral position, pillows should be placed:**

a. Under the head, at the back, and between the legs. ( )

b. Under the head, upper arm, upper legs, and at the back. ( )

c. Under the upper arm, at the back, and between the legs. ( )

d. Under the head, upper arm, and between the legs. ( )

**30. An appropriate nursing care for managing mechanical load is:**

a. Elevating the head of the bed at 30o. ( )

b. Cleaning soil. ( )

c. Use lubricants and lotions. ( )

d. Turning position. ( )

**31. An appropriate nursing activity to reduce friction is:**

a. Elevating the head of the bed greater than 30o. ( )

b. Lifting patient without dragging. ( )

c. Placing pressure-relieving devices. ( )

d. Elevating the head of bed at 90o. ( )

**32. The nursing care for reducing shearing force is.**

a. Elevating the head of bed < 30o. ( )

b. Elevating the head of bed at 30o. ( )

c. Elevating the head of bed at 60o. ( )

d. Elevating the head of bed at 90o. ( )

**33. Exercise prevents pressure ulcers through:**

a. Improving blood circulation. ( )

b. Providing energy. ( )

c. Preventing tissue damage. ( )

c. Promoting soothing effect. ( )

**34. The most serious complication of a pressure ulcer is:**

a. Marjolijn ulceration. ( )

b. Blister formation. ( )

c. Sepsis. ( )

d. Gas gangrene. ( )

**Practice Regarding Preventing Pressure Ulcers.**

| **Not Done** | **Done** | **Items** |  |
| --- | --- | --- | --- |
|  |  | I observe how other nurses assess the risk factors |  |
|  |  | I identify common contributing factors to PU |  |
|  |  | I do a skin assessment |  |
|  |  | I use a risk assessment scale |  |
|  |  | I document all data |  |
|  |  | I assess and provide management of pain |  |
|  |  | I perform skin care as routine work |  |
|  |  | I place the pillow under the patient’s leg |  |
|  |  | I use water-filled gloves under the patient’s leg |  |
|  |  | I use or advise caregivers to use creams or oils |  |
|  |  | I pay more attention to pressure points |  |
|  |  | I perform lab tests |  |
|  |  | I provide vitamins and food |  |
|  |  | I monitor a protein and calorie diet |  |
|  |  | I avoid dragging |  |
|  |  | I always use a special mattress |  |
|  |  | I avoid massage |  |
|  |  | I avoid using a donut–shaped (ring) cushion |  |
|  |  | I turn the patient's position every two hours. |  |
|  |  | I put pillows under the patient’s leg and ankle |  |
|  |  | I always attend seminars. |  |
|  |  | I give advice to the patient or caregiver. |  |

**Nurses' attitude toward pressure ulcer prevention.**

| **Strongly Disagree** | **Disagree** | **Neutral** | **Agree** | **Strongly Agree** | **Items** |  |
| --- | --- | --- | --- | --- | --- | --- |
|  |  |  |  |  | All patients are at potential risk of developing pressure ulcers. |  |
|  |  |  |  |  | Pressure ulcer prevention is time-consuming for me to carry out. |  |
|  |  |  |  |  | In my opinion, patients tend not to get as many pressure ulcers nowadays. |  |
|  |  |  |  |  | In my practice, I do not need to concern myself with pressure ulcer prevention. |  |
|  |  |  |  |  | Pressure ulcer treatment is a greater priority than pressure ulcer prevention. |  |
|  |  |  |  |  | Continuous assessment of patients will give an accurate account of their pressure ulcer risk. |  |
|  |  |  |  |  | Most pressure ulcers can be avoided. |  |
|  |  |  |  |  | I am less interested in pressure ulcer prevention than in other aspects of care. |  |
|  |  |  |  |  | My clinical judgment is better than any available pressure ulcer risk assessment tool. |  |
|  |  |  |  |  | In comparison with other areas of care, pressure ulcer prevention is a low priority for me. |  |
|  |  |  |  |  | Pressure ulcer risk assessment should be regularly carried out on all patients during their stay in the hospital. |  |

**Nurses Burnout Scale**

|  | **Burnout** | Never | Rarely | Sometimes | Often | Always |
| --- | --- | --- | --- | --- | --- | --- |
| Exhaustion | | | | | | |
|  | I feel mentally exhausted* |  |  |  |  |  |
|  | At the end of the day, I find it hard to recover my energy |  |  |  |  |  |
|  | 4. I feel physically exhausted |  |  |  |  |  |
| Mental distance | | | | | | |
|  | I struggle to find any enthusiasm for my work |  |  |  |  |  |
|  | I feel a strong aversion towards my job |  |  |  |  |  |
|  | I’m cynical about what my work means to others |  |  |  |  |  |
| Cognitive impairment | | | | | | |
|  | I have trouble staying focused |  |  |  |  |  |
|  | I have trouble concentrating |  |  |  |  |  |
|  | I make mistakes because I have my mind on other things |  |  |  |  |  |
| Emotional impairment | | | | | | |
|  | I feel unable to control my emotions |  |  |  |  |  |
|  | I do not recognize myself in the way I react emotionally |  |  |  |  |  |
|  | I may overreact unintentionally |  |  |  |  |  |

The end
